# RosettaEPR: Rotamer Library for Spin Label Structure and Dynamics

**DOI:** 10.1371/journal.pone.0072851

**Published:** 2013-09-05

**Authors:** Nathan S. Alexander, Richard A. Stein, Hanane A. Koteiche, Kristian W. Kaufmann, Hassane S. Mchaourab, Jens Meiler

**Affiliations:** 1 Center for Structural Biology, Vanderbilt University, Nashville, Tennessee, United States of America; 2 Department of Chemistry, Vanderbilt University, Nashville, Tennessee, United States of America; 3 Department of Molecular Physiology and Biophysics, Vanderbilt University, Nashville, Tennessee, United States of America; Max Planck Institute for Polymer Research, Germany

## Abstract

An increasingly used parameter in structural biology is the measurement of distances between spin labels bound to a protein. One limitation to these measurements is the unknown position of the spin label relative to the protein backbone. To overcome this drawback, we introduce a rotamer library of the methanethiosulfonate spin label (MTSSL) into the protein modeling program Rosetta. Spin label rotamers were derived from conformations observed in crystal structures of spin labeled T4 lysozyme and previously published molecular dynamics simulations. Rosetta’s ability to accurately recover spin label conformations and EPR measured distance distributions was evaluated against 19 experimentally determined MTSSL labeled structures of T4 lysozyme and the membrane protein LeuT and 73 distance distributions from T4 lysozyme and the membrane protein MsbA. For a site in the core of T4 lysozyme, the correct spin label conformation (Χ_1_ and Χ_2_) is recovered in 99.8% of trials. In surface positions 53% of the trajectories agree with crystallized conformations in Χ_1_ and Χ_2_. This level of recovery is on par with Rosetta performance for the 20 natural amino acids. In addition, Rosetta predicts the distance between two spin labels with a mean error of 4.4 Å. The width of the experimental distance distribution, which reflects the flexibility of the two spin labels, is predicted with a mean error of 1.3 Å. RosettaEPR makes full-atom spin label modeling available to a wide scientific community in conjunction with the powerful suite of modeling methods within Rosetta.

## Introduction

**Figure 1:**
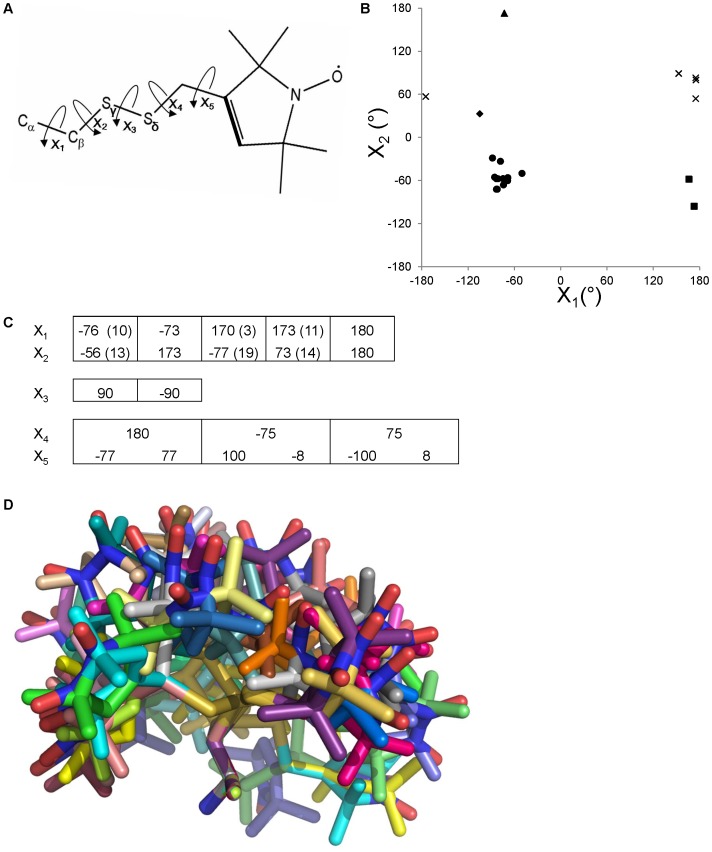
Rotamer library generation. A.) Designation of the five rotatable bonds in the methanethiosulfonate spin label (MTSSL) side chain. X_1_ is defined with the backbone nitrogen atom. X_5_ is defined by the doubly bonded carbon atom (bold) [27], [32]. B.) Combinations of MTSSL X_1_ and X_2_ angles observed in T4 lysozyme crystallographically. {m, t} = ▴; {m,m} = •; {t,m} = ▪; {t,p} = x. The diamond (♦) denotes what is observed at core site mutant L118; excluding this point, four groups of X_1_ and X_2_ combinations are observed. C.) Combinations of X angles used in the MTSSL rotamer library. X_1_ and X_2_ are correlated and there are five combinations possible. X_3_ is not correlated with any other X angle and there are two possible conformations of X_3_. X_4_ and X_5_ are correlated such that for each X_4_ angle, there are two possible X_5_ angles. Enumerating the possible combinations gives 5×2×3×2 = 60 total possible rotamer conformations. Numbers in parentheses give standard deviations, if available. D.) After removing conformations with internal clashes, 54 rotamers remain in the library.

Electron paramagnetic resonance (EPR) can be applied to both large and membrane proteins (MPs). Thereby, EPR opens an avenue to study the structure and dynamics of proteins which are often difficult to study with X-ray crystallography or nuclear magnetic resonance (NMR) [1], [2]. Pulsed EPR, specifically double electron-electron resonance (DEER), in conjunction with site directed spin labeling (SDSL) allows specific inter-residue distances to be routinely measured up to 60 Å [3]–[5] and can reach up to 80 Å [6], [7]. The limitation of EPR in its application to protein structure determination is that the distances are measured between unpaired electrons in the nitroxide group of the spin label side chain. The most widely used methanethiosulfonate spin label (MTSSL) projects from the backbone of the protein. It has five rotatable bonds (Χ_1_–Χ_5_) with an a priori unknown conformation between the Cα of the protein backbone and the unpaired electron at the midpoint of the N-O bond. Without the knowledge of the spin label conformation, it is difficult to directly relate the distance between the unpaired electrons to a distance between its anchor points on the protein backbone. This task becomes even more challenging in solvent exposed positions on the protein surface with little spatial restriction. Here the spin label will adopt an ensemble of conformations with comparable free energies [8] (Figure 1 A). In result, a broad distance distribution for the unpaired electrons is observed in the EPR measurement [3], [9], [10].

Previous computational methods have been developed to determine correct spin label conformations [11], [12] and structurally interpret EPR distance distributions [13] within a protein environment. While generally successful, these techniques relied upon computationally intensive molecular dynamics, Monte Carlo searches, or combinations of the two, in order to effectively sample the necessary conformational space available to the spin label probe. The algorithms focused on the local environment around the spin label assuming a rigid protein backbone in order to make the calculation computationally tractable but potentially missing preferred rotamers.

Libraries of likely conformations of spin labels (rotamers) have been previously applied for explicit modeling of MTSSL. A rotamer is a likely side chain conformation with a specific set of chi angles derived from statistical analysis of the Protein Data Bank (PDB) [14]. An initial library of 62 rotamers [7] was expanded to 98 [15] and then to approximately 200 rotamers [10] in order to capture the allowable conformational space of the spin label. The rotamer libraries in the latter study were derived from molecular dynamics calculations of spin label flexibility. These methods accurately predicted a) conformations of MTSSL seen in experimentally determined soluble structures and b) measured distance distributions between spin labels in doubly mutated soluble proteins.

Further, a knowledge-based potential was introduced [16], [17] which, in combination with coarse-grained potentials and sparse EPR distance restraints, can be used to determine protein topology. Instead of a full-atom model of the spin label, it converts the experimental spin label distance into a probability distribution of Cβ distances. While efficient in determining the protein fold with RosettaEPR, the potential lacks detail needed for high-resolution structure refinement.

The objective of the present work is to extend RosettaEPR with a full-atom representation of the spin label that aligns with the Rosetta “rotamer” approach for rapid sampling of protein side chain degrees of freedom [18]. The ability of Rosetta to recover native rotamers has been demonstrated for protein structure prediction [16], [19], [20] and protein design [21]. The present study extends the amino acid rotamer libraries used by Rosetta to include MTSSL. The rotamer library for MTSSL is derived from the experimentally and computationally observed correlated preferences of the side chain dihedral angles. Consequently, the library consists of only 54 conformations. The incorporation of MTSSL into RosettaEPR enables modeling of the spin label in a wide range of Rosetta protocols such as full-atom refinement [20], [22] and membrane protein modeling [23]–[25]. After initial placement of the spin label rotamer, the Rosetta full-atom potential enables sampling of off-rotamer conformations thereby limiting the number of initial rotamers needed. RosettaEPR optimizes all other protein side chains and backbone degrees of freedom in parallel [19], allowing backbone and neighboring side-chain perturbations caused by the spin label to be captured. RosettaEPR makes the technology readily available to the EPR community through RosettaCommons free non-commercial licensing.

The current study details the development of Rosetta’s MTSSL rotamer library and demonstrates: a) Rosetta’s ability to sample MTSSL conformations experimentally observed in 19 structures of the soluble protein T4 lysozyme and the membrane protein LeuT; b) Rosetta’s ability to recover the experimental probability distribution for a measured EPR distance in T4 lysozyme and the membrane protein MsbA; and c) the unbiased cross-validation of the cone model parameters [16], [17].

## Results

### MTSSL Rotamer Library

Sixteen structures of T4 lysozyme with single MTSSL mutations [26]–[29], and one with a double MTSSL mutation [29], have been determined experimentally by X-ray crystallography, allowing 21 low energy conformations of the MTSSL side chain to be observed (Table S1). The labels in the double mutant K65/R80 to MTSSL are structurally independent and do not interact [29], so for the purposes of this study will be considered separate individual single mutants. Two single MTSSL mutations of LeuT have been determined by X-ray crystallography (Table S2) [30]. Here, the convention of Lovell et al. [31] is used to denote Χ_1_ and Χ_2_ angles; Χ_1_ = 0 when S_γ_ eclipses the backbone nitrogen (Figure 1 A). Additionally, “m”, “p”, and “t” indicate dihedral angles of −60°, +60°, and 180°, respectively. Tombolato et al. [32] defines Χ_5_ as S_δ_ – C – C = C, which is the convention used here (Figure 1 A). Although most of the mutations are on exposed helical sites, crystal structures for one core position [28] and exposed loop residues [26] have been determined. This experimental knowledge base provides the necessary foundation for building a rotamer library for MTSSL.

Note that a rotamer not only captures likely conformations for all Χ-angles but also their respective interdependences, i.e. how likely a certain combination of Χ-angles is observed. The relatively small number of spin label conformations observed experimentally forbids a statistical analysis of all interdependences between Χ_1_–Χ_5_, because many experimental structures lack information on Χ_4_- and Χ_5_-angles. Assuming just three conformations for each of the Χ_1,2,4,5_-angles and two for Χ_3_, 162 conformations need to be considered. While some of those can be excluded for internal clashes, the number of possible conformations is still much larger than the 21 experimental conformations available. Approximately 500 experimental structures resolving all Χ-angles would be needed to build a complete rotamer library from a knowledge base. Therefore, we follow a hybrid approach deriving likely (Χ_1_, Χ_2_) combinations from experimental structures. Possible conformations for Χ_3_ are taken from quantum chemical studies [32] which agree closely with crystallographic data. Χ_3_ is decoupled from Χ_1_ and Χ_2_, i.e. all combinations of Χ_3_ with (Χ_1_, Χ_2_) pairs will be considered. Combinations of Χ_4_ and Χ_5_ are derived from quantum chemical studies [32], since these Χ-angles are resolved in only four experimental structures. We expect to update this rotamer library as additional experimental structures of the spin label become available.

Only four (Χ_1_, Χ_2_) combinations of m, t, and p have been experimentally observed: {m, m}, {m, t}, {t, p}, and {t, m} (Figure 1 B). One conformation of MTSSL observed in the core of the protein [28] is excluded from consideration from the rotamer library because it cannot be classified into the “m”, “t”, or “p” categories described above. It was observed only once, so it remains unclear if this conformation represents a low energy state of the spin label in isolation or is induced by packing interaction in the protein core. While a single conformation is insufficient to perform the statistical analysis needed for creation of a rotamer, Rosetta relaxation protocols will be capable of modeling off-rotamer conformations starting from one of the rotamers provided (read below). Quantum chemical calculations have shown that also the {t, t} conformation, not yet seen in any experimental structure, is sterically allowed for sites on an exposed poly-alanine helix [32]. Therefore, the {m, m}, {m, t}, {t, p}, {t, m}, and {t, t} conformations are represented in the current rotamer library as the average angle observed for each pair (Figure 1 C, Table S3).

Χ_3_ is experimentally and computationally observed to adopt an angle of ±90°, independent of Χ_1_ and Χ_2_. As a result, both states will be considered for each of the five sets of Χ_1_ and Χ_2_ angles (Figure 1 C). In the instance where Χ_3_ is 53°, the crystal structure reveals several favorable contacts in the crystal lattice that presumably overcome the unfavorable energy of the distortion [29]. This Χ_3_ angle was not considered in the rotamer library.

Χ_4_ and Χ_5_ have been observed in only five and four of the crystal structures, respectively. Due to the small sample size for (Χ_4_,Χ_5_) combinations, the values predicted from quantum chemical calculations are used [32]. The calculations predict a correlation between Χ_4_ and Χ_5_, where the highest probability conformers are: a) when Χ_4_ is 180°, Χ_5_ is ±77°b) when Χ_4_ is −75°, Χ_5_ is either −8° or +100°c) when Χ_4_ is +75°, Χ_5_ is either 8° or −100° (Figure 1 C). Key surface interactions of mutant T115^100^ (mutation of residue 115 to MTSSL; superscripts denote temperature) and core packing of mutant L118 cause the Χ_4_ and Χ_5_ values to be 76° and 98° for T115^100^ and 54° and 107° for L118 [28]. These values were not considered in the rotamer library, though if additional structures show these to be frequently observed conformations, they will be added.

Taking into account all combinations of the Χ angles, there are 60 possible rotamers (5×2×3×2 = 60). However, these 60 rotamers include some conformations which contain intramolecular clashes. After removing conformations with internal atomic clashes and minimization to alleviate minor clashes (please see Experimental procedures section for more details), 54 rotamers form the MTSSL rotamer library for RosettaEPR (Figure 1 D).

### Ability of Rosetta to Recover Experimentally Observed Spin Label Conformations

MTSSL mutants of the soluble T4 lysozyme protein (17 mutants) and the LeuT membrane protein (2 mutants) were used to demonstrate the ability of Rosetta to recover conformations of spin labels experimentally observed. For each mutant, approximately 1,000 independent relaxation trajectories were conducted and the percentage of models finding the experimentally observed Χ angles was calculated (Table 1, Table 2). Values within ±30° were considered correct [27]. The percentages are computed such that preceding Χ angles must be correct before a more distal angle can be counted as correct. For example, Rosetta predicts the crystallized Χ_1_ angle of T4 lysozyme mutant T151^100^ 100% of the time and predicts both, the experimental Χ_1_ and Χ_2_ angles, 51% of the time correctly. If there is more than one empirical conformation, a model rotamer is counted as correct if it matches any experimentally observed conformations.

**Table 1:** Percentage of Rosetta relaxation trajectories that recover experimental X angles in T4 lysozyme.

T4 Lysozyme Mutant	Environ.	X_1_	X_2_	X_3_	X_4_	X_5_
L118	core	99.8	99.8	99.8	99.8	99.8
S044_C_	surface	67.6	0.1			
R080	surface	100.0	100.0			
A082	surface	61.8	61.8	61.8		
T115^100^	surface	79.3	63.6	63.6	2.4	2.4
T115^298^	surface	95.0	19.9			
T115/R119A	surface	24.4	1.1			
R119	surface	74.9	1.2			
V131^100^	surface	100.0	99.9			
V131^291^	surface	99.9	99.2			
T151^100^	surface	100.0	51.0			
T151^291^	surface	98.6	78.1			
**Mean**		83.4	56.3	75.1	51.1	51.1
A041	crystalcontact	60.2	56.0	46.3		
S044_A_	crystalcontact	13.7	0.0	0.0	0.0	0.0
S044_B_	crystalcontact	0.8	0.0	0.0	0.0	0.0
K065	crystalcontact	34.0	33.2	0.0		
V075	crystalcontact	1.2	1.2	1.2	0.2	

Superscripts indicate the temperature at which the crystal X-ray data was collected.

Subscripts indicate the component of the crystallographic asymmetric unit as given in the PDB file.

Blank boxes indicate those X angles were not resolved crystallographically.

**Table 2:** Percentage of Rosetta relaxation trajectories that recover experimental X angles in LeuT.

LeuTMutant	Environ.	X_1_	X_2_	X_3_	X_4_	X_5_
F177	Surface	10.0	2.5	0.2	0.2	0.2
I204	Surface	77.8	60.2	3.9	2.8	2.8
**mean**		43.9	31.4	2.1	1.5	1.5

Excluding crystal contact sites, Rosetta samples the correct rotamer for each of the fourteen structures. Χ_1_ and Χ_2_ are correctly predicted in nine out of fourteen cases with at least 50% frequency. In seven out of twelve cases for T4 lysozyme, Rosetta recovers all experimentally observed Χ angles at least 50% of the time. On average for the fourteen mutants of T4 lysozyme and LeuT, recovery of experimentally observed Χ_1_ and Χ_2_ occurs in 53% of sampling trajectories (Figure S1, Figure S2).

**Figure 2:**
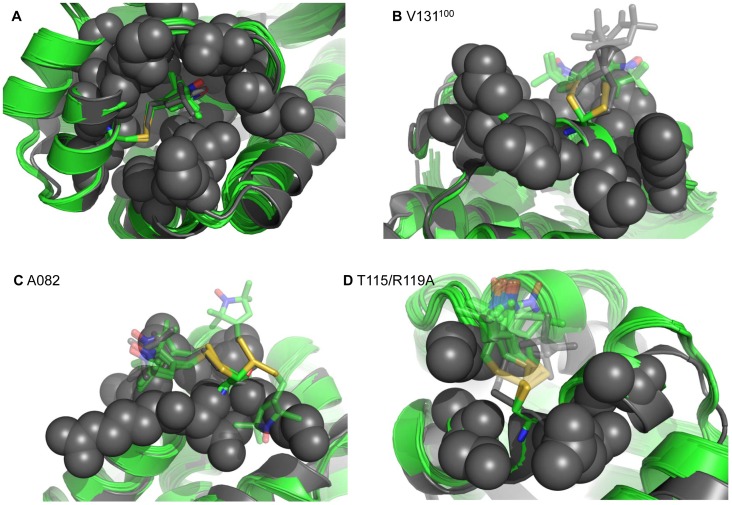
Recovery of experimentally observed spin label conformations. Ten best scoring Rosetta models (green) overlayed with the crystal structure (grey) for four examples of MTSSL mutated sites on T4 lysozyme. Crystallographically observed X angles are shown solid, while atoms and X angles not experimentally seen are translucent. A) Rosetta’s ability to recover a crystallographically observed spin label conformation at buried site 118 in T4 lysozyme. Spheres are used to indicate the buried nature of the site. B.) Two conformations of X_1_ and X_2_ were experimentally observed for single mutant site V131^100^. Rosetta models frequently sample these two conformations of X_1_ and X_2_. C) X_1_, X_2,_ and **X**_3_ were experimentally observed for mutant A082. Several of the top ten conformations by Rosetta score sample these X angles, while other conformations are also sampled with a lower frequency. D) One conformation of X_1_ and X_2_ was observed for mutant T115/R119A. None of the ten best Rosetta models by score sample the experimental conformation.

In the only mutant at a buried site L118, Rosetta recovers the experimentally observed Χ angles 99.8% of the time. The pocket in which the spin label resides greatly restricts the number of possible non-clashing conformations (Figure 2 A). The crystallized Χ_1_ and Χ_2_ angles are distorted from the expected values due to the steric constraints of the pocket. In spite of the Χ_1_ and Χ_2_ not being in the rotamer library, Rosetta’s potentials are able to accurately drive the spin label to adopt the correct conformation starting from one of the rotamers.

Surface mutants allow the spin label the possibility to adopt more conformations than core sites due to the reduced number of surrounding residues. As a result, Rosetta finds often multiple low-energy conformations for spin labels. This results in three scenarios: a) Rosetta almost exclusively (greater than 75%) samples the experimental Χ angles for four out of the thirteen surface mutants (Figure 2 B); b) Rosetta sometimes (approximately 50%) samples the observed rotamers for two out of the thirteen surface mutants (Figure 2 C).; and c) Rosetta seldom (less than 20%) samples the experimental conformations for seven out of the thirteen surface mutants (Figure 2 D). Three of these seven cases involve the instances where Χ_1_–Χ_5_ are observed, making it difficult for Rosetta to find the experimental conformation for all the degrees of freedom. In the other four cases, only Χ_1_ and Χ_2_ are observed so it is difficult to determine what, if any, interactions lead Rosetta to frequently differ from the experimentally observed conformations.

With the exception of one mutant (A041), Rosetta is unable to successfully recover the observed Χ angles at crystal contact sites. The Χ angles of A041 are recovered with approximately the same frequency as the one of the surface mutants. Of the other spin labels placed at crystal contact sites, Rosetta samples all experimental Χ angles of only V075 and does so only 0.2% of the time (see Discussion).

### Ability of Rosetta to Recover Experimental Distance Distributions

Fifty-eight EPR measured distance distributions have been collected for the T4 lysozyme protein [4], [16], [33], including twelve new measurements. Additionally, nine EPR distance measurements of less than 70 Å in transmembrane segments of the membrane protein MsbA in the apo-open and ten in the AMP-PNP bound state have previously been collected [34]. These data provide an opportunity to test Rosetta’s ability to recover experimental distance distributions. Such distributions can be roughly characterized as an average distance (μ_EPR_) and a standard deviation (σ_EPR_). Each spin labeled double mutant model for T4 lysozyme and MsbA was subjected to 2000 and about 1000 independent relaxation trajectories within Rosetta, respectively. The mean (μ_Rosetta_) and standard deviation (σ_Rosetta_) of the inter-spin label distance was then calculated for the best 200 and 100 models according to Rosetta score for T4 lysozyme and MsbA, respectively. Filtering for the best 10% by score has been successfully employed with Rosetta in the past [19], [35], [36], and preliminary analysis indicated this as being appropriate for the current work as well. Four T4 lysozyme double mutants (131/154, 131/151, 140/147, 116/131) were excluded from analysis because, for each, the standard deviation of the experimental measurement (4.0, 8.0, 7.0, 10.0, respectively) is greater than 50% of the measured distance (6.5 Å, 9.0 Å, 13.0 Å, 19.0 Å, respectively). This could result from them not falling entirely within the applicable DEER range. The midpoint of the N-O bond was used as the location of the unpaired electron [10].

**Figure 3:**
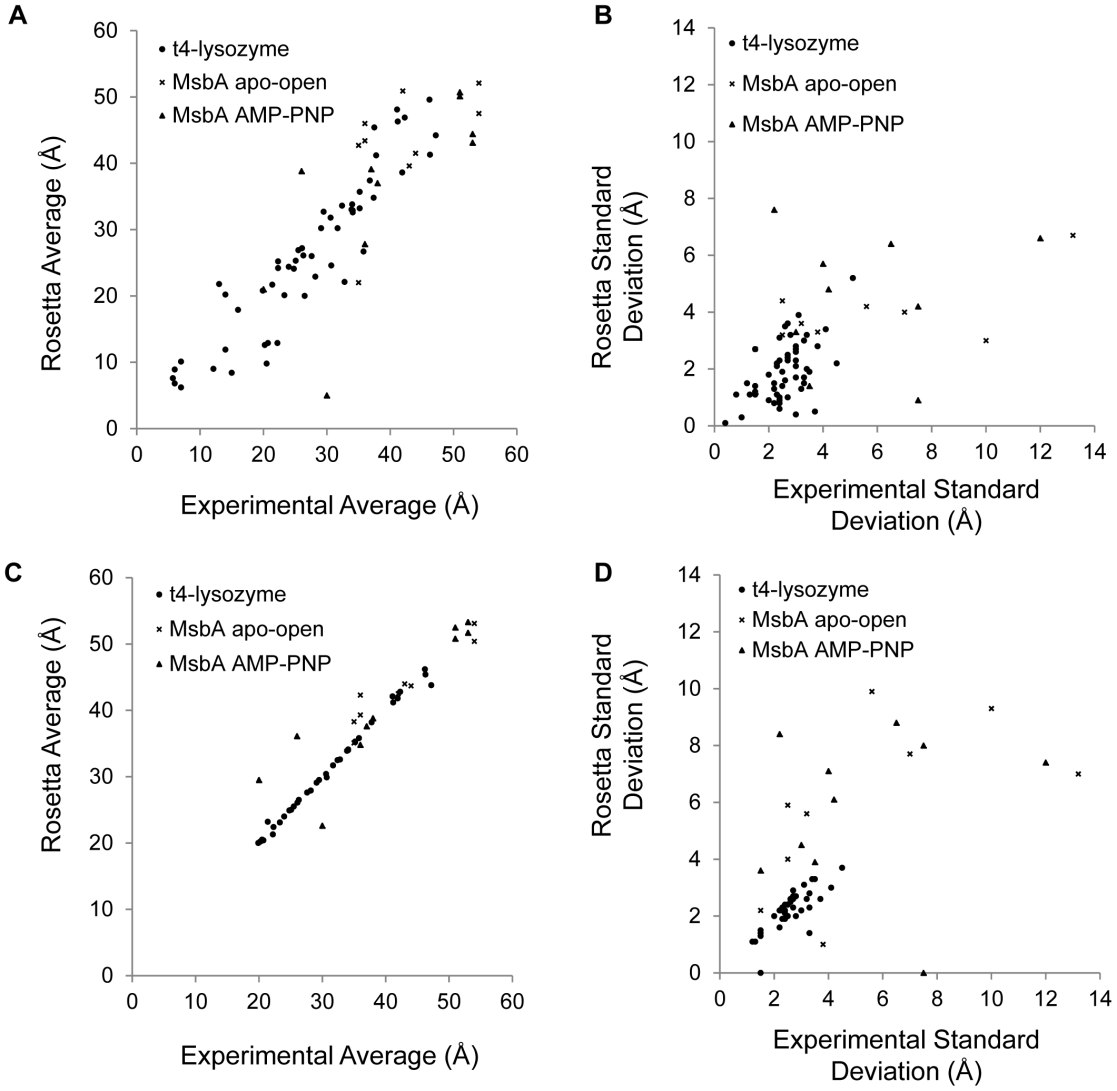
Recovery of experimentally observed distance distributions. Plots of the average distance and standard deviation of ensembles of T4 lysozyme and MsbA double mutant distance distributions sampled by Rosetta versus the experimentally determined mean and standard deviation. A and B) The ensembles of the best 200 (for T4 lysozyme) and 100 (for MsbA) models by Rosetta score. C) and D) The ensembles of Rosetta models determined by fitting the models to the experimental distance distributions.

Across all distance distributions, Rosetta achieves a mean absolute error (MAE, see Experimental Procedures) for μ_Rosetta_ versus μ_EPR_ of 4.4 Å (Table 3, Figure S3, Figure S4, Figure S5). This is compared to a MAE of 6.1 when Cβ atoms are used to approximate the position of the spin label, indicating that Rosetta is able to provide additional, more accurate information compared to a simple Cβ approximation for the spin label. On the T4 lysozyme dataset, the MAE for μ_Rosetta_ compared to μ_EPR_ is 3.5 Å (Figure 3 A *circles*, Table S4). This is an improvement over simply using Cβ atoms, which gives a MAE of 5.7 Å (Table S7). For the MsbA dataset, the MAE for μ_Rosetta_ compared to μ_EPR_ is 6.8 Å (Figure 3 A *crosses*, Table S5) and 7.0 Å (Figure 3 A *triangles*, Table S6) for the apo open and AMP-PNP bound states, respectively. This offers a 0.4 Å improvement in MAE for the AMP-PNP bound state when compared to using Cβ distances (Table S8, Table S9).

**Table 3:** Statistical measures of how well Rosetta recovers µ _EPR_ and σ_EPR_ for T4 lysozyme and MsbA double mutants.

	μ_Rosetta_			σ_Rosetta_		
	MAE	RMSD	R	MAE	RMSD	R
T4 lysozyme	3.5	4.5	0.92	0.9	1.1	0.56
MsbA apo-open	6.8	7.6	0.53	2.5	3.5	0.67
MsbA AMP-PNP bound	7.0	10.2	0.72	2.6	3.5	0.24
Combined*	4.4	6.1	0.89	1.3	2.0	0.58
Combined** ^Cβ^	6.1	7.1	0.93	2.7	3.2	0.55

* values calculated from all T4 lysozyme and MsbA double mutants for spin label distances.

** values calculated from all T4 lysozyme and MsbA double mutants for Cβ distances.

The standard deviation of the distribution of distances determined in an EPR distance measurement (σ_EPR_) indicates the breadth of conformations of MTSSL and of the backbone sampled by the ensemble of labeled proteins present during the experiment. The standard deviation for the distribution of distances determined by Rosetta (σ_Rosetta_) for all double mutants achieves a MAE to σ_EPR_ of 1.3 Å (Table 3). The MAE of σ_Rosetta_ across the T4 lysozyme dataset is 0.9 Å (Figure 3 B *circles*, Table S4), compared to MAE of 2.4 Å if Cβ are used to approximate the spin label position (Table S7). For the MsbA datasets in the apo-open and AMP-PNP bound states, σ_Rosetta_ has an MAE of 2.5 Å (Figure 3 B *crosses*, Table S5) and 2.6 Å (Figure 3 B *triangles*, Table S6), respectively. Compared to using Cβ approximations, σ_Rosetta_ is better in MAE by 0.6 Å and 1.1 Å for the apo-open and AMP-PNP bound states of MsbA, respectively (Table S8, Table S9).

Broad distributions of distances measured for MsbA in the apo-open and AMP-PNP bound states make it difficult for Rosetta to recover μ_EPR_ and σ_EPR_ as accurately as is done for T4 lysozyme. The average σ_EPR_ over the nineteen MsbA measurements is 5.3 Å as opposed to 2.6 Å for the T4 lysozyme distributions, and the distributions can contain multiple peaks spread out over a wide range of distances. This is indicative of significant backbone fluctuations independent of spin label conformation. Rosetta’s difficulty with reproducing μ_EPR_ and σ_EPR_ for MsbA therefore arises a) due to the difficulty in summarizing broad complex distributions into a mean and standard deviation and b) because the relaxation protocol is not expected to produce large backbone changes. Additionally, one must be cautious when utilizing long distances as there potentially can be more uncertainty in longer distances due to issues such as background correction and data quality, than in shorter distances. Therefore, the accuracy of RosettaEPR for MsbA must be considered within the context of the error associated with the long distance measurements.

### RosettaEPR Samples within all Experimental Distance Probability Distributions

For thirty-eight of the T4 lysozyme [4], [16], [33] and all nineteen of the MsbA [34] experimental double mutant EPR measurements, distance probability distributions were available. These data sets allow the models generated for each double mutant by Rosetta to be used in a fitting procedure to determine if, out of these models, an ensemble can be formed that accurately reproduces the experimental distance distribution. This experiment was designed to assert whether the current limitations are in sampling (conformations needed not in the ensemble) or scoring (conformations needed rank not best). Since the rotamer library is derived from limited data from crystal structures and supplemented with data from molecular dynamics, such an experiment is important to exclude the possibility of too limited sampling.

The 2000 models for each double mutant of T4 lysozyme and the top 1000 models by Rosetta score for each mutant of MsbA in the apo-open and AMP-PNP bound states were used to find an ensemble reproducing the corresponding distance distribution. After this procedure and across all double mutants, the MAE of the average distance calculated from the Rosetta ensemble, 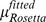, compared to μ_EPR_ is 1.1 Å (Table 4). For T4 lysozyme double mutants, the MAE of 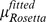 is 0.3 Å (Table S10), compared to 3.5 Å for the top 10% of models according to Rosetta score. The MAE of 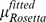 for the apo-open and AMP-PNP bound states of MsbA drops to 2.1 Å (Table S11) and 3.3 Å (Table S12), compared to 6.8 Å and 7.0 Å, respectively.

**Table 4:** Statistical measures of how well Rosetta recovers µ _EPR_ and σ_EPR_ for T4 lysozyme and MsbA double mutants after selecting relaxed structures to match the experimental distance distributions.

	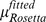			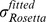		
	MAE	RMSD	R	MAE	RMSD	R
T4-lysozyme	0.3	0.7	1.00	0.4	0.6	0.80
MsbA apo-open	2.1	2.9	0.95	2.5	3.1	0.59
MsbA AMP-PNP bound	3.3	5.0	0.90	3.0	3.8	0.14
Combined*	1.1	2.5	0.97	1.2	2.1	0.63

* values calculated from all T4 lysozyme and MsbA double mutants for spin label distances.

The standard deviation calculated from ensembles of Rosetta models selected to fit the corresponding distance distribution, 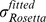, for T4 lysozyme double mutants achieves an MAE of 0.4 Å to σ_EPR_ compared to 0.9 Å for the top 10% of models according to Rosetta score. For double mutants of MsbA, the MAE of 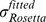 in the apo-open and AMP-PNP bound states are 2.5 Å and 3.0 Å, respectively, which is not an improvement over selecting models strictly by score.

Instead of attempting to summarize the shape of distance distributions with µ and σ, using a measure to compare the entire distribution (cumulative Euclidean distance, see Experimental Procedures) can more accurately describe the improvement in Rosetta’s ability recover the distributions of T4 lysozyme and MsbA after fitting (Figure S6, Figure S7, Figure S8). For T4 lysozyme double mutants, the error in the ensembles of Rosetta models is reduced by an average of 87% (Table S13). Although 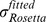 was not sensitive to improvements in the agreement between Rosetta and experimental distance distributions for MsbA, comparison of the distributions show an average reduction in error of 62% (Table S14) and 54% (Table S15) for the apo-open and AMP-PNP bound states, respectively. Over all double mutants, the error is reduced by an average of 77% with an average ensemble size of 18 relaxed structures.

### Validation of Implicit Spin Label Cone Model Parameters

The introduction of a full-atom representation of MTSSL within Rosetta allows the explicit description of the ensemble of conformations accessible to spin labels attached to various sites on a protein. The previously published spin label cone-model implicitly described the ensemble of conformations using uniform parameters applied to all sites [16], [17]. It defined an effective position for the spin label (SL_ef_) as the positional average of all possible spin label locations as it projects from the protein backbone. The “cone model” assumes the allowable spin label positions are contained within a cone with a defined opening angle (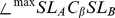 = 90°; Figure S9 A), which corresponds to the maximum observed angle between any two spin labels with vertex C_β_. The cone model also assumes the cone is oriented at a random angle with respect to the protein backbone (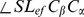 = 120°, Figure S9 B). Lastly, as a trigonometric result of 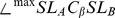 and the length of the spin label tether (8.5 Å), the cone model defines a distance from the C_β_ to the SL_ef_ (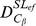 = 6 Å, Figure S9 C).

The Rosetta rotamer library was used to explicitly compute the cone model parameters and compare with the original assumptions. Residues at 162 exposed sites on the primarily α-helical T4 lysozyme (PDBid 2LZM) and β-strand chitinase (PDBid 2CWR) [37] proteins were computationally mutated to create 162 single spin labeled mutants. Each of these mutants was subjected to 500 independent Rosetta relaxation trajectories in order to obtain an ensemble of allowable spin label conformations at each site.

The parameters calculated from the Rosetta ensembles are comparable to the original cone model parameters (Table 5). The distribution of 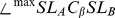 values shows a mean 103° with standard deviation of 50° (Figure S10 A). For 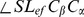, the Rosetta distribution shows a mean of 111° and a standard deviation of 63° (Figure S10 B). The values of 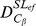 sampled by Rosetta have a mean of 6.3 Å and a standard deviation of 1.2 Å (Figure S10 C).

**Table 5:** Comparison of the parameters used by the cone model [16] of a spin label and the values recovered by the rotamer library.

Parameter	Cone Model	Rosetta Explicit Spin Label Model	
		Mean	Standard Deviation
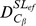 (Å)	6.0	6.3	1.2
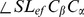 (°)	120	111	63
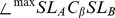 (°)	90	103	50

**Figure 4:**
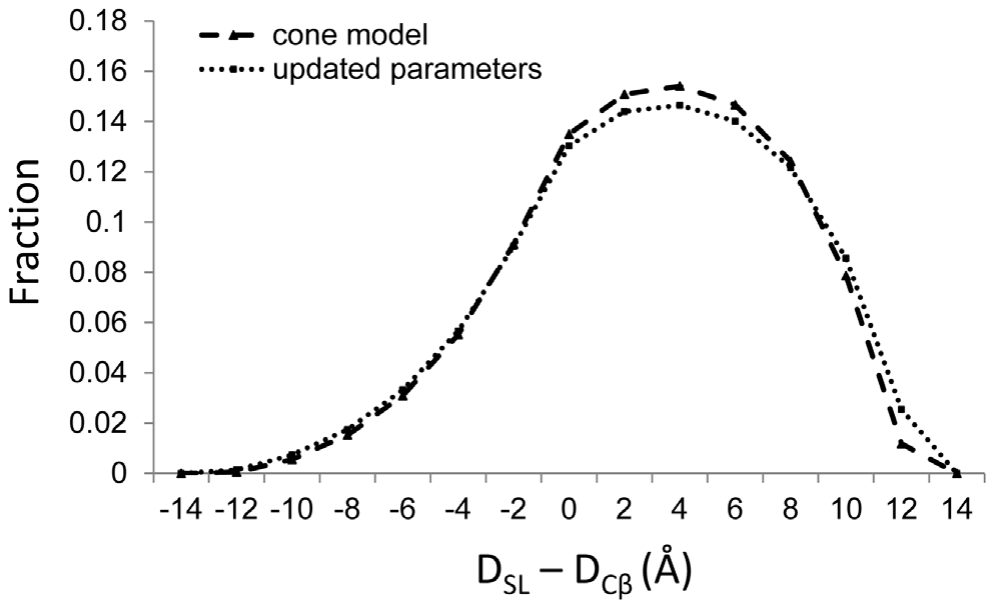
Comparison of cone model statistics. Statistics on the frequency with which D_SL_–D_Cβ_ is observed for the initial [16] cone model parameters (cone model) and the updated parameters calculated from RosettaEPR (updated parameters). D_SL_ is a distance between two spin labels, where each has been randomly oriented and approximated by the corresponding cone model parameters. D_Cβ_ is the distance between the C_β_ atoms of the residues containing the spin labels. The frequency is given on the y-axis as the fraction of observed D_SL_–D_Cβ_ values falling within a given bin.

Figure 4 displays a comparison of D_SL_–D_Cβ_ statistics for the initial cone model [16] with an updated cone model computed using the currently calculated parameters. D_SL_ is a distance between two spin labels, as approximated by the cone model. D_Cβ_ is the distance between the C_β_ atoms of the residues containing the spin labels. With the increased length of 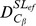 and the decreased 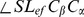 compared to initial values, there is an increased fraction of D_SL_–D_Cβ_ values between 10 Å and 12 Å. However, the small difference in the curves demonstrates the robustness of the cone model to small deviations in the parameters.

## Discussion

The RosettaEPR spin label rotamer library leverages experimentally observed and computationally predicted correlations between Χ angles of MTSSL. A rotamer library reduces the side chain Χ-angle search space in order to produce a biologically probable conformation. Such efficiency allows RosettaEPR to sample in parallel with the spin label all other protein side chains and backbone degrees of freedom, rather than being restricted to a rigid protein structure. All-atom refinement of the protein structure allows determination of off-rotamer spin label conformations and offers the potential to sample small, local backbone and side chain structural perturbations caused by the spin label. However, in practice, it will be difficult for the energetic contributions of the spin label to overcome energetic barriers of large conformational changes such as those leading to unstructured residues (Figure S11). Correctly capturing inter-side-chain surface interactions is also a very challenging task (Figure S12).

### RosettaEPR Rotamer Library Combines Experimentally Determined Spin Label Conformations with Quantum Chemical Calculations

The present knowledge-base of experimentally observed MTSSL conformations is small. Therefore, the current rotamer library supplements experimentally observed (X_1_, X_2_) combinations with computationally predicted Χ_3–5_ angles. Specifically, the (X_1_, X_2_) {t, t} rotamer has not yet been experimentally observed but was added to the rotamer library based on quantum chemical calculations [32]. Χ_3_ was considered to be ±90°which is in agreement with both, experimental values and quantum chemical calculations [32]. Conformations for Χ_4_ and Χ_5_ were determined experimentally only four times for the soluble T4 lysozyme protein. This rotamer library therefore relies on quantum chemical calculations alone [32] for Χ_4_ and Χ_5_. As additional crystal structures of MTSSL become available, especially for membrane proteins, the rotamer library will be extended to take into account an expanded experimental knowledge-base. The immediate advantage of full-atom verification of EPR experiments outweighs the current limits of the knowledge-based rotamer library.

### RosettaEPR Spin Label Library is Robust enough for Use in a Wide Range of Modeling Protocols of Proteins

Compared with a systematic search of larger rotamer libraries, the RosettaEPR rotamer library is limited to a relatively small number of 54 discrete conformers which maximizes efficiency of the conformational search and enables parallel optimization of additional protein degrees of freedom. However, it is important to ensure that having a small number of rotamers is not a limiting factor in the sampling ability of RosettaEPR. Therefore, this approach is balanced by sampling off-rotamer conformations in all-atom refinement protocols. Further, Rosetta systematically samples close-to-rotamer conformations by varying (X_1_, X_2_) by one standard deviation. The number of spin label rotamers aligns with the number of rotamers seen for large amino acid side chains (Arg, Lys 81 rotamers [38]), which have been demonstrated to be sufficient for atomic-detail structure determination [16], [19], [35], [39]. The success of the approach is demonstrated by a) recovery of the off-rotamer experimental conformation of T4 lysozyme mutant L118 (Figure 2 A), b) Rosetta’s ability to sample all experimentally observed conformations of MTSSL in soluble T4 lysozyme and the membrane protein LeuT, and c) the ability of the Rosetta models to accurately fit the experimental EPR distance distributions (Figure 3 C and 3 D). Only as additional experimental data becomes available will the robustness of RosettaEPR be able to be exhaustively tested.

### RosettaEPR Samples Experimentally Observed Spin Label Conformations on the Surface and in the Protein Core for Soluble and Membrane Proteins

RosettaEPR samples all experimentally observed conformations of MTSSL at core and surface sites at least in some trajectories. However, RosettaEPR also samples alternative conformations sometimes with a higher frequency and superior energy to the experimentally observed conformation. A combination of reasons is expected to contribute to this result: a) the spin label samples multiple and additional conformations of similar free energy in solution that are not observed in the crystal. This notion is supported by the frequent uncertainty in reconstructing spin labels on the surface of proteins as displayed by lack of coordinates beyond X_3_. b) The RosettaEPR energy function ranks different conformations of the spin label incorrectly with respect to each other. This is expected on the protein surface given the close free energy of such conformations, the approximations inherent to the pair-wise decomposable Rosetta energy function [18], and the lack of specific treatment of electrostatic interactions the nitroxide group might engage the protein in.

It is important to note that, due to limited experimental data, the crystal structures are used both in the generation and testing of the rotamer library. Therefore, the ability of RosettaEPR to sample the conformations in the crystal structures contained in the rotamer library is not surprising. Another limitation in our approach is that the labeling sites are almost exclusively on exposed helices. However, the ability of RosettaEPR to select for the experimentally observed conformation is an important finding. The current results demonstrate that Rosetta has the accuracy to distinguish between different spin label conformations and select for the experimentally observed conformations. As more spin label crystal structures become available further testing of RosettaEPR will be carried out.

RosettaEPR poorly samples the experimental conformations of MTSSL at crystal contact sites. Each protein component of the asymmetric unit was relaxed in Rosetta independently, i.e. not in the presence of the other copies in the crystal. Therefore, such performance is expected because the spin label conformations are significantly influenced by non-biologically relevant crystal contact interactions that are not present in examination of the rotamers in RosettaEPR [26]–[29].

### RosettaEPR Reproduces Specific Dynamics Seen for Spin Labels

RosettaEPR achieves an MAE of 4.4 Å for predicting experimental EPR distances. This compares favorably to usage of the C_β_ distances as an approximation for the spin label (MAE = 6.1 Å). The cone model fits the difference between spin label distance and C_β_ distance to a set of experimental data [16], [17]. It minimizes the RMSD between experimental and predicted distance to 4.7 Å which is comparable to the explicit treatment of the spin label in RosettaEPR. This indicates the power of a simple linear correlation between spin label and C_β_ distances. However, the cone model inherently assumes the same conformational sampling, σ, for all spin labels independent of labeling site which is also represented by the standard deviation of the distance difference distribution (4.7 Å). The standard deviation of the experimental distance distributions are reproduced much more closely by the full-atom representation of the spin label with a RMSD of 2.0 Å. Thereby, explicit treatment of the spin label provides information on the actual conformational sampling of MTSSL.

By selecting ensembles of models from RosettaEPR specifically to reproduce experimental EPR distance probability distributions, the accuracy of RosettaEPR is further improved. RosettaEPR can sample within all of the experimental distance probability distributions. This indicates the range of sampling with the rotamer library is not the limiting factor in RosettaEPR’s ability to reproduce spin label dynamics. For double mutants where sampling within the experimental probability distribution is infrequent, a more accurate scoring function could focus sampling to produce smoother, more accurate fits to the distributions.

### Comparison with Previous Methods

RosettaEPR recovers native Χ_1_ and Χ_2_ of MTSSL with a frequency similar to Rosetta’s ability to recover arginine and lysine Χ_1_ and Χ_2_. Over a dataset of 129 proteins, Rosetta recovered native Χ_1_ and Χ_2_ of arginine and lysine 60–65% of the time [40]. Though this is a slightly higher percentage than observed for MTSSL, the fraction of exposed positions in the MTSSL dataset is large, which would account for the reduced accuracy of RosettaEPR.

RosettaEPR’s rotamer recovery is slightly less accurate than the side chain prediction method SCWRL4 [39] in recovery for Χ_1_ and Χ_2_ (70%) and Χ_1_–Χ_4_ (36%) in arginine and lysine side chains across buried and exposed sites in 379 protein structures. However, as Χ_1_ and Χ_2_ recovery is calculated for arginine and lysine at increasingly exposed positions, the performance of SCWRL4 more closely aligns with RosettaEPR’s Χ_1_ and Χ_2_ recovery for MTSSL. This is important because thirteen of the fourteen MTSSL single mutants at non-crystal contact sites occur at surface positions. A similar scenario is seen for the MtsslWizard method [41]. The MtsslWizard only takes into account Van der Waals clashing to determine allowable spin label conformations. Therefore, as the labeled site becomes more exposed or specific interactions are important, the accuracy decreases.

In T4 lysozyme, single mutants A082 and L118 were used for the study of an MTSSL rotamer library [10]. This study was also successful in predicting the experimentally observed conformations at these sites. However, for L118, the population of rotamers predicted to be buried within the cavity as observed in the experimental structure is 99.8% for RosettaEPR versus 52% for the previous study. Without additional experimental data, it is difficult to determine which is more accurate.

A previous attempt at recovering the average distance of an EPR double mutant measurement have a reported mean error of 3.0 Å over twenty-seven distances measured in troponin C, the troponin complex and the KcsA channel [13]. Rosetta EPR achieves MAE of 4.4 Å over all seventy-three EPR distances for T4 lysozyme and MsbA, and 3.5 Å for fifty-eight T4 lysozyme distances specifically. Differences in accuracy are mitigated by the differences in the protein systems and size of the datasets.

A more recent analysis was applied to a subset of the the T4L distances reported here [41]. This analysis compared a rotamer approach as implemented in MMM [10] to an unrestricted search approach, MTSSLWizard [41]. The results indicated that the search approach was better than the rotamer approach at obtaining the average distance. In none of the studies was the widths of the distributions from the modeling compared to the experimental widths carried out.

We have applied the free and open-source packages MMM and MTSSLWizard to the full set of T4L distances reported here (Table S16) and compared them to the results for RosettaEPR (Table S17). We find that MTSSLWizard is better by 0.5 Å MAE than MMM and RosettaEPR at finding the center of the distance distribution (Figure S13). Examination of the widths of the distributions indicates that MMM and MTSSLWizard exhibit essentially the same width of the distribution (∼3 Å) regardless of the actual experimental width. RosettaEPR is the only method that exhibits a correlation between the modeled and experimental width of the distribution (Figure S14).

The utility of fitting an ensemble of structures to EPR distance data has been demonstrated for the transmembrane domain IX of the Na^+^/proline transporter PutP of *Escherichia coli* [15]. This single transmembrane span has a helix-loop-helix motif. MTSSL rotamers and backbone ψ, φ were varied to produce an RMSD of 1.00 Å of the models to experimental mean distances. This compares favorably to the 0.7 Å RMSD achieved by RosettaEPR over the thirty-eight T4 lysozme distributions and 2.5 Å when all fifty-seven distributions (T4 lysozyme and MsbA) are considered.

### Verification of Cone Model Parameters

The distribution of 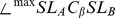 observed indicates that the width of the spin label conformational ensemble (the opening angle of the cone) can vary widely across different sites on a protein. The original cone model parameter of 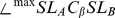 = 90° falls within one standard deviation of the 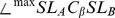 distribution average. The distribution of 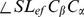 obtained by Rosetta indicates that the ensemble can be tilted closely towards the backbone, indicative of the spin label hugging the surface of the protein. Given the hydrophobic nature of the MTSSL side chain, it is likely the spin label would exhibit such behavior. The average 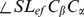 value calculated from RosettEPR of 111° matches closely with the original parameter of 120°. The distance between the effective spin label position and the corresponding C_β_, 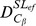, was originally proposed in the cone model to be 6.0 Å. The distribution obtained by RosettaEPR indicates that 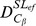 value is on average slightly longer at 6.3 Å. The 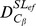 is related to 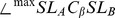 as an increasing width of the ensemble will produce a decreasing 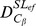. The fact that the average 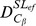 is slightly longer than what would be expected given the average 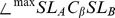 is due to the population of MTSSL ensembles with a small width.

Overall we find the cone model parameters accurate within the error of the experiment. It is apparent that while the cone model rather accurately captures distances, experimental distance deviations are not adequately represented with a unified model. Through the full-atom description of spin labels during structure prediction, this study overcomes one critical limitation of the cone model. The cone model was derived by observing spin label distances over many independent experiments. Spin label pairs in very different structural and dynamical states were folded into a single probability distribution. This probability distribution encompasses uncertainty over the precise conformation of the spin label and its dynamics, convoluting both contributions. Its allowable distance range is therefore inherently too wide. The model is very effective in medium-resolution modeling due to its speed and due to omitting explicit modeling of side chains – an approach that is widely used at this stage. At the same time it reaches its limitations in atomic-detail refinement of the models – for example restraints were not employed for atomic-detail refinement in our previous research on de novo folding of proteins from EPR restraints [16], [17].

Potentially, RosettaEPR could yield insight into the environmental factors that determine the disorder of the spin label at a site. Such a scenario could occur as the database of crystallographically observed spin label conformations grows, allowing for an improved scoring function describing the interactions of the nitroxide with its environment. With an accurate description of the nitroxides behavior, a refined cone model would allow for the quick verification of a putative model or structure.

## Conclusion

RosettaEPR can recover and sample experimentally observed conformations of the MTSSL spin label on single mutants of T4 lysozyme and the membrane protein LeuT. RosettaEPR’s ability to reproduce EPR distance distributions has not previously been demonstrated. The MAE of 4.4 Å for T4-lysozyme distances means that each spin label in the distance is accurate to an average of 2.2 Å. Modeling MTSSL at this level of accuracy makes important steps towards atomic-detail refinement of protein structures based on experimental EPR distance restraints, making RosettaEPR a powerful tool for investigating the structure and dynamics of proteins.

## Experimental Procedures

### Development of MTSSL Rotamer Library

The non-canonical methanesulfonothioate spin label residue was created in the Molecular Operating Environment [42]. The Pymol Molecular Graphics System [43] was then used to create 60 rotamers taking into account all the possible combinations of the canonical Χ angles as elaborated in the Results section. The potential energy of each rotamer was calculated for use as an indicator of which rotamers contained intramolecular clashes. The potential energy was calculated in MOE using the “Potential” function with the default MMFF94× force field. The rotamers were sorted by energy. Ten rotamers were determined to have clashes because a large increase in potential energy (54.9%) for the most energetically favorable of the ten rotamers separated them from the other 50 rotamers. Outside of these ten rotamers, the largest potential energy increase was 10%. The ten rotamers were subject to energy minimization in MOE using the “MM” function in an attempt to rescue each rotamer in the event that small changes to the Χ angles could relieve the clash. After minimization, the potential energy of eight of the ten rotamers was minimized into the regime of the other 50 rotamers. In addition to a reduction in potential energy, the eight minimized rotamers were also filtered by the amount of change in each Χ angle such that no Χ angle changed by more than 30°. Four of the eight rotamers met this criterion. As a result, the total rotamer library contains 54 conformations of MTSSL.

### Single Mutant MTSSL Conformational Sampling

Each of the crystal structures of T4 lysozyme singly labeled with MTSSL were downloaded from the Protein Data Bank (PDB) [44]. The PDB accession identifiers (PDB IDs) are 2IGC, 2OU8, 2OU9, and 2NTH [28], 2Q9D and 2Q9E [27], and 1ZYT, 2CUU, 3G3V, 3G3W, and 3G3× [26] (See Table S1 for identification of the mutant for each PDB file). Mutants R080, R119, K065, and V075 [29] were not available to download from the PDB website. Therefore, the single mutants for these were computationally created from the T4 lysozyme crystal structure with PDB ID 2LZM [45]. In order to create the cys-less sequence [46], which was used for these four single mutant crystal structures, cysteine residues 54 and 97 were computationally mutated to threonine and alanine, respectively. All computational mutations were done using the Rosetta Fixed Backbone Design application [21]. Each crystallized protein structure, including those involving crystal contacts, was relaxed (see below) in Rosetta individually without the presence of any other crystallographic subunits. The starting protein structures were subjected to 1000 independent relaxation trajectories in Rosetta, which were then used for analysis on Rosetta’s ability to recover experimentally observed conformations.

For single MTSSL mutants of LeuT, the two experimental structures downloaded were 3MPN and 3MPQ ([30]). These structures were relaxed by Rosetta in 1015 trajectories.

### Double Mutant MTSSL Conformational Sampling

A pseudo wild type starting structure was created as described above whereby cysteine residues 54 and 97 of PDB ID 2LZM were computationally mutated to threonine and alanine, respectively. Next, structures for 58 double mutants were created from this pseudo wild type starting structure. Forty-six of these mutants have been previously described [4], [16], [33] with twelve new double mutants (Figure S15). All computational mutations were done using the Rosetta Fixed Backbone Design application. Each of these fifty-eight double mutants was subjected to 2000 independent relaxation trajectories in Rosetta. For each relaxation trajectory, the distance between the final conformations of the two spin labels was calculated, where the unpaired electron is taken to be at the midpoint of the N-O bond. The set of distances from the top 200 of models by Rosetta score was used as the distance distribution for each mutant, and compared against the corresponding experimental distance distributions. Double mutants 131/154, 131/151, 140/147, and 116/131 were excluded from analysis because the standard deviation of the distance measurement was determined to be greater than 50% of the distance. The experimental distance distributions for double mutants 119/128, 119/131, 123/131, and 140/151 were reanalyzed for this study using Tikhonov regularization [9], producing means and standard deviations of the distributions which differ slightly from the originally published values [16].

Nineteen previously published EPR distances measured in the transmembrane region of MsbA [34] were used for this study. Computational double mutants were created from PDB ID 3B60 [47] for the AMP-PNP closed state and from the full-atom structure of the open state provided from [34]. Coordinates of the full-atom open state structure will be provided upon request. Cysteine residues 88 and 315 were mutated to alanine, resulting in the pseudo wild type used for creating computational double MTSSL mutants. All double mutants were relaxed at least 1000 times in Rosetta and the top 100 models by Rosetta score were used as the distance distribution for each mutant.

Three statistical values are used to compare Rosetta to EPR experiment. The mean absolute error (MAE) is calculated as 
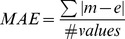
, where *m* is the model value and *e* is the experimental value. The root mean square deviation (RMSD) is calculated as RMSD = 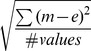. The correlation coefficient (R) is also used for comparison of Rosetta to experiment.

### Rosetta Relaxation and Computational Mutant Protocols

The standard Rosetta refinement protocol [19], [20] was used to relax the T4 lysozyme protein structures and determine MTSSL conformations. For MsbA and LeuT, the relaxations took place using the membrane specific potentials of Rosetta [23]. During relaxation all side chains are repacked and small perturbations of the backbone occur. This means that the starting conformations of side chains do not impact the final rotamers chosen. A single Rosetta relaxation trajectory takes about 15 minutes on an Intel Xeon W3570 3.2 GHz processor for T4 lysozyme. Please see Experimental Procedures S1 for the specific command line flags used.

The fixed backbone design application of Rosetta was used to introduce MTSSL at desired sites in the benchmark proteins. The protocol does not allow any backbone optimization and all other side chains were held fixed in their native conformation. So, only the conformation of the specific mutated residue was optimized, which was sufficient because the mutants later underwent Rosetta relaxation. The application takes approximately one minute to run on an Intel Xeon W3570 3.2 GHz processor. Please see Experimental Procedures S1 for specific command line flags used.

### Fitting of Rosetta Generated Ensembles to Experimental EPR Distance Distributions

Fifty-seven experimental EPR distance distributions analyzed by Tikhonov regularization were used as the dataset for finding Rosetta generated ensembles that give spin-label to spin-label distance distributions similar to experiment: thirty-eight from T4 lysozyme and nineteen from MsbA. For each T4 lysozyme double mutant, all 2000 relaxation models were possible constituents of the matching sub-ensemble. For MsbA, the top 1000 models according to Rosetta score were available for fitting. A Monte Carlo process of adding or removing models and allowing only favorable moves was used to determine the matching sub-ensembles. Agreement between the EPR measured and Rosetta recovered distance distributions calculated from the sub-ensemble was measured by the cumulative Euclidian distance 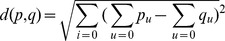 [48], where *p* and q give the probability of a given distance bin, and *u* and *i* are iterations over the distance bins. This value 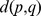 is normalized by the number of bins summed over, N, such that 
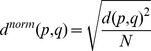
.

### Derivation of Implicit Spin Label Cone Model Parameters

The primarily alpha-helical T4 lysozyme pseudo-wild type starting structure and the primarily beta-strand chitinase (PDB ID 2CWR [37] were used as the basis to determine the implicit model parameters. Single mutations introducing MTSSL were computationally created for the two proteins at residues having a neighbor count [49] less than ten. 63 and 99 sites met this neighbor count criteria for T4 lysozyme and 2CWR, respectively. Each of these single mutants was subjected to 500 independent relaxation trajectories in Rosetta.

For each single mutant, the effective spin label position, SL_ef_, was calculated as the average of all the observed positions of the N-O bond midpoints on the nitroxide moiety of the spin label. In order to determine SL_ef_, the backbone C_α_, H_α_, C, N, and CB atoms of the spin label were used to superimpose the 500 structures for each mutant. Superimposition was done using the “fit” command in Pymol. SL_ef_ was then calculated for each single mutant along with the corresponding 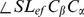 and 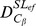 parameters. Also, 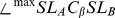 was determined for each single mutant after superimposition, by calculating all pairwise 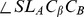 for the 500 models and finding the maximum value observed.

These updated parameters for the cone model were then used to simulate spin-spin label distances, D_SL_, in multiple proteins. 4379 single chains from soluble proteins filtered by PISCES [50] for not more than 25% sequence identity and resolution of at most 2.0 Å were used to calculate the distances. These spin label distances, D_SL_, were then compared to the distance between the C_β_ atoms of the residues containing the spin labels, D_Cβ_. A histogram describing the difference between D_SL_ and D_Cβ_, was then calculated.

## Supporting Information

Figure S1 **All experimentally observed MTSSL Χ_1_ and Χ_2_ angles for single mutant models of T4 lysozyme.** Squares with dark lines indicate the experimentally observed Χ_1_ and Χ_2_ values ±30°. Squares with light grey lines indicate combinations of Χ_1_ and Χ_2_ which are contained in the rotamer library. The frequency with which combinations of Χ_1_ and Χ_2_ which are sampled by Rosetta for each single mutant are given according to grey scale with white areas never being sampled and darker areas being sampled more frequently.
(TIF)

Figure S2 **All experimentally observed MTSSL Χ_1_ and Χ_2_ angles for single mutants of LeuT.** Squares with dark lines indicate the experimentally observed Χ_1_ and Χ_2_ values ±30°. Squares with light grey lines indicate combinations of Χ_1_ and Χ_2_ which are contained in the rotamer library. The frequency with which combinations of Χ_1_ and Χ_2_ which are sampled by Rosetta for each single mutant are given according to grey scale with white areas never being sampled and darker areas being sampled more frequently.
(TIF)

Figure S3 **Heat maps for 58 double mutants of T4 lysozyme showing Gaussian distributions given by experimentally measured mean and standard deviation parameters compared with distance distributions recovered by Rosetta from the top 200 models according to Rosetta score.** *Experimental* distance distributions are the *top bar* and Rosetta distributions are the bottom bar for each pair of heat maps. Distances are given in Angstroms, and the probability of observing a distance is defined by grayscale. Mutants 131/154, 131/151, 140/147, 116/131 were excluded from statistical analysis but are shown here for completeness.
(TIF)

Figure S4 **Heat maps for 9 double mutants of MSBA in the apo-open state showing Gaussian distributions given by experimentally measured mean and standard deviation parameters compared with distance distributions recovered by Rosetta from the top 100 models according to Rosetta score.** *Experimental* distance distributions are the *top bar* and Rosetta distributions are the bottom bar for each pair of heat maps. Distances are given in Angstroms, and the probability of observing a distance is defined by grayscale.
(TIF)

Figure S5 **Heat maps for 10 double mutants of MSBA in the AMP-PNP bound state showing Gaussian distributions given by experimentally measured mean and standard deviation parameters compared with distance distributions recovered by Rosetta from the top 100 models according to Rosetta score.** *Experimental* distance distributions are the *top bar* and Rosetta distributions are the bottom bar for each pair of heat maps. Distances are given in Angstroms, and the probability of observing a distance is defined by grayscale same as Figure S4.
(TIF)

Figure S6 **Agreement between experimental distance probability distributions and an ensemble of Rosetta models fitted to the experimental distribution for 38 double mutants of t4-lysozyme.** Curves show the integral of the probability up to a given distance.
(TIF)

Figure S7 **Agreement between experimental distance probability distributions and an ensemble of Rosetta models fitted to the experimental distribution for double mutants of MSBA in the apo-open state.** Curves show the integral of the probability up to a given distance.
(TIF)

Figure S8 **Agreement between experimental distance probability distributions and an ensemble of Rosetta models fitted to the experimental distribution for double mutants of MSBA in the AMP-PNP bound state.** Curves show the integral of the probability up to a given distance.
(TIF)

Figure S9
**Visual description of the three parameters that define the cone model and their relation to the full-atom representation of the spin label.** The effective spin label position, SL_ef_, is the average position of the midpoint of the N-O bond vector. In B.) and C.) the SL_ef_ position is represented as a red sphere. A.) 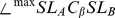 is the opening angle of the cone and is calculated as the widest angle observed between two MTSSL conformations obtained from Rosetta. B.) 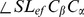 is the angle defined by the C_α_, C_β_, and SL_ef_ positions, and gives information on the allowable tilt angles of the cone. C.) 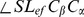 is the distance from the C_β_ to the SL_ef_ position.(TIF)

Figure S10
**Distributions of the parameters that define the “cone model” as determined by Rosetta using the rotamer library full-atom representation of MTSSL.** Shown are the frequencies with which given values of A.) 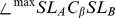 B.) 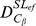, and C.) 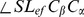 are observed by Rosetta at 162 singly labeled MTSSL sites on primarily alpha-helical and beta-strand proteins.(TIF)

Figure S11 **Relaxation of T4-lysozyme single mutant L118R1A starting from non-mutant crystal structure.** The crystal structure of the T4-lysozyme single mutant L118R1A (PDB ID 2NTH) is shown in magenta. The pseudo-wildtype structure described in “Experimental Procedures” based on the crystal structure with PDB ID 2LZM was computationally mutated to contain a spin label at site 118 and relaxed ten times. The ten structures are shown. Residues 108–113 are unstructured in 2NTH, allowing space to accommodate the spin label. The corresponding helical residues in 2LZM remain structured after relaxation and the spin label is necessarily placed in an orientation different from that seen in 2NTH in order to avoid backbone clashes.
(TIF)

Figure S12 **T4-lysozyme single mutant T115^100^R1A is the only non-crystal contact surface site where all five Χ angles have been observed.** The structure has PDB ID identifier 2IGC and is shown in black. Out of the 1000 relaxation trajectories, twenty-four structures have the correct conformation of the spin label. The surrounding residues within 5 Å of the spin label are shown in sticks.
(TIF)

Figure S13 **Plots of the average spin label distance from T4-lysozyme spin labeled double mutant distance distributions predicted by RosettaEPR, MMM, and MTSSLWizard compared to the experimental average distance measured by EPR.**
(TIF)

Figure S14 **Plots of the standard deviation of spin labeled double mutant T4-lysozyme distance distributions predicted by RosettaEPR, MMM, and MTSSLWizard compared to the experimental standard deviation measured by EPR.**
(TIF)

Figure S15 **For spin labeled double mutants of T4-lysozyme, background-corrected normalized echo decay traces from DEER measurements with corresponding distance distributions obtained from Tikhonov regularization.**
(TIF)

Table S1 **Experimentally determined MTSSL conformations for single mutants of T4-lysozyme.**
(DOC)

Table S2 **Experimentally determined MTSSL conformations for single mutants of LeuT.**
(DOC)

Table S3 **Combinations of Χ_1_ and Χ_2_ leading to the combinations contained in the rotamer library.**
(DOC)

Table S4 **The average (μ) and standard deviation (σ) of inter-spin label distance distributions for double mutants of T4 lysozyme.**
(DOC)

Table S5 **The average and standard deviation of inter-spin label distance distributions for double mutants of MSBA in the apo open state.**
(DOC)

Table S6 **The average and standard deviation of inter-spin label distance distributions for double mutants of MSBA in the AMP-PNP bound state.**
(DOC)

Table S7 **Using Cβ atoms to approximate the position of spin labels in T4 lysozyme.**
(DOC)

Table S8 **Using Cβ atoms to approximate the position of spin labels in MSBA in the apo open state.**
(DOC)

Table S9 **Using Cβ atoms to approximate the position of spin labels in MSBA in the AMP-PNP bound state.**
(DOC)

Table S10 **Analysis of the best ensemble of Rosetta models fitted to the experimental distance probability distributions for T4 lysozyme.**
(DOC)

Table S11 **Analysis of the best ensemble of Rosetta models fitted to the experimental distance probability distributions for MSBA in the apo open state.**
(DOC)

Table S12 **Analysis of the best ensemble of Rosetta models fitted to the experimental distance probability distributions for MSBA in the AMP-PNP bound state.**
(DOC)

Table S13 **Disagreement to experimental distance distributions of models selected by score and fitting in T4 lysozyme double mutant models.**
(DOC)

Table S14 **Disagreement to experimental distance distributions of models selected by score and fitting for MsbA in the apo-open state double mutant models.**
(DOC)

Table S15 **Disagreement to experimental distance distributions of models selected by score and fitting for MsbA in the AMP-PNP bound state double mutant models.**
(DOC)

Table S16 **The MMM and MTSSLWizard average (μ) and standard deviation (σ) of inter-spin label distance distributions for double mutants of T4 lysozyme.**
(DOC)

Table S17 **Descriptions of the disagreement between prediction and experiment for the average distance and standard deviation of distance distributions from RosettaEPR, MMM, and MTSSLWizard.**
(DOC)

Experimental Procedures S1 **Command lines used for Rosetta protocols.**
(DOC)
