# Supplementary figures and images for: RosettaEPR: Rotamer Library for Spin Label Structure and Dynamics
Source: PLoS One. 2013 Sep 5;8(9):e72851. doi: 10.1371/journal.pone.0072851 (PMC3764097; doi:10.1371/journal.pone.0072851)

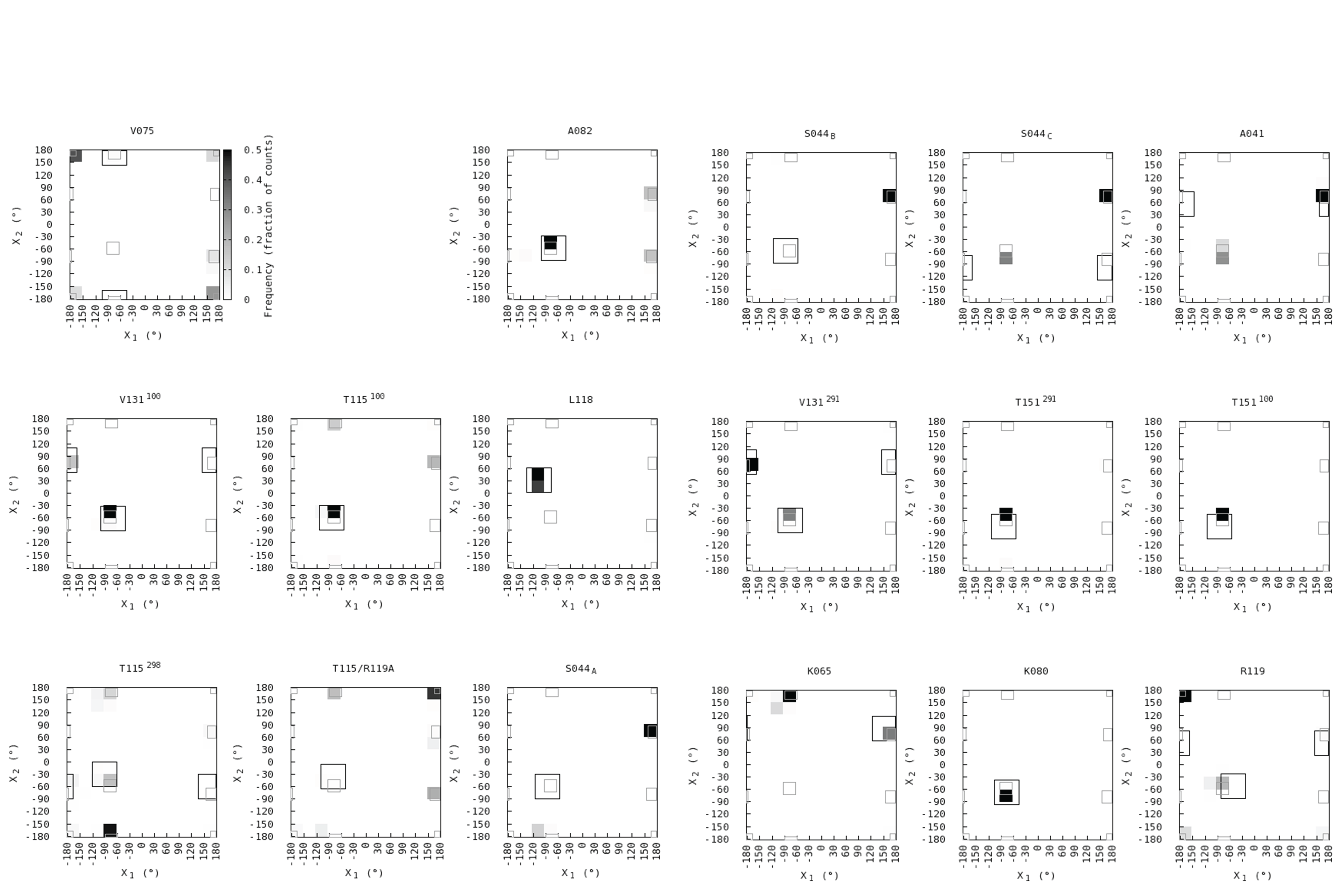

Supplement: Figure S1 — All experimentally observed MTSSL Χ1 and Χ2 angles for single mutant models of T4 lysozyme. Squares with dark lines indicate the experimentally observed Χ1 and Χ2 values ±30°. Squares with light grey lines indicate combinations of Χ1 and Χ2 which are contained in the rotamer library. The frequency with which combinations of Χ1 and Χ2 which are sampled by Rosetta for each single mutant are given according to grey scale with white areas never being sampled and darker areas being sampled more frequently. (TIF) [file pone.0072851.s001.tif]

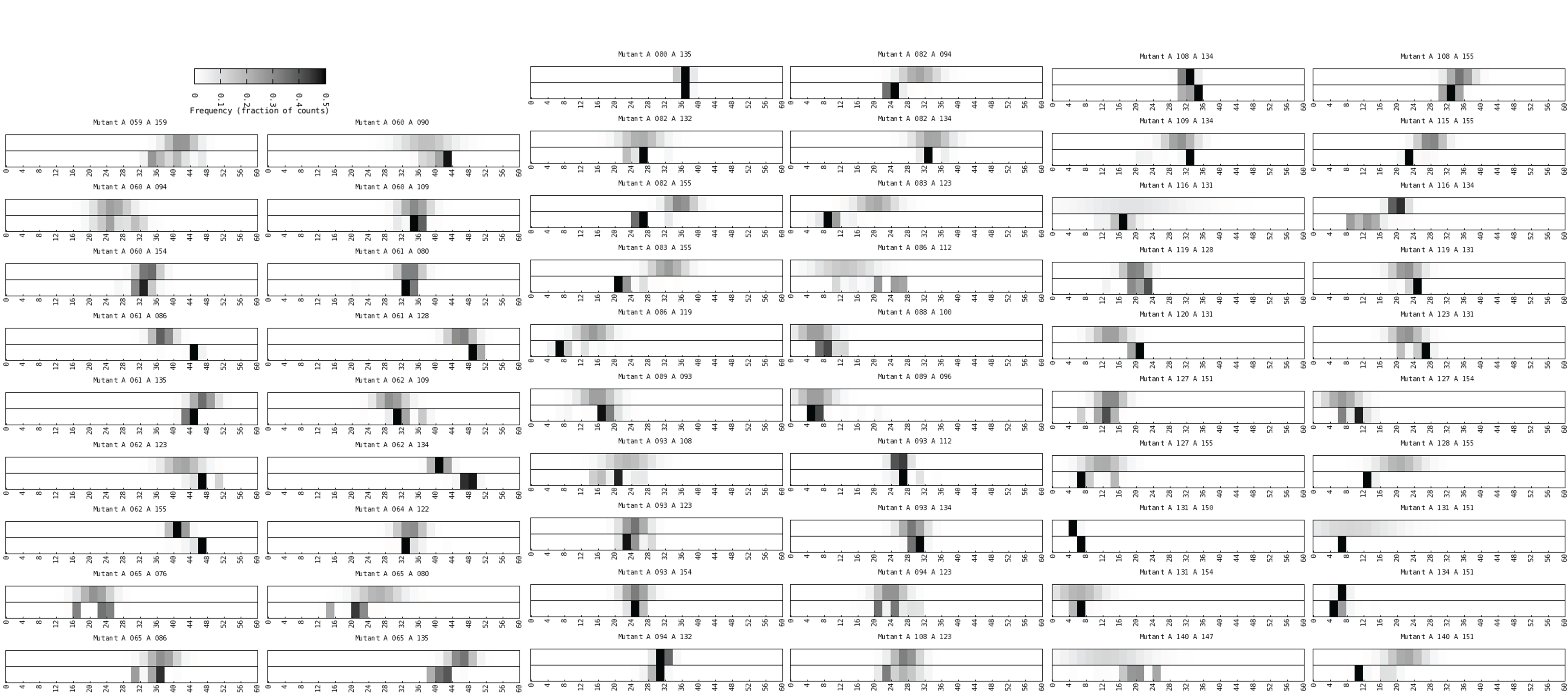

Supplement: Figure S3 — Heat maps for 58 double mutants of T4 lysozyme showing Gaussian distributions given by experimentally measured mean and standard deviation parameters compared with distance distributions recovered by Rosetta from the top 200 models according to Rosetta score. Experimental distance distributions are the top bar and Rosetta distributions are the bottom bar for each pair of heat maps. Distances are given in Angstroms, and the probability of observing a distance is defined by grayscale. Mutants 131/154, 131/151, 140/147, 116/131 were excluded from statistical analysis but are shown here for completeness. (TIF) [file pone.0072851.s003.tif]

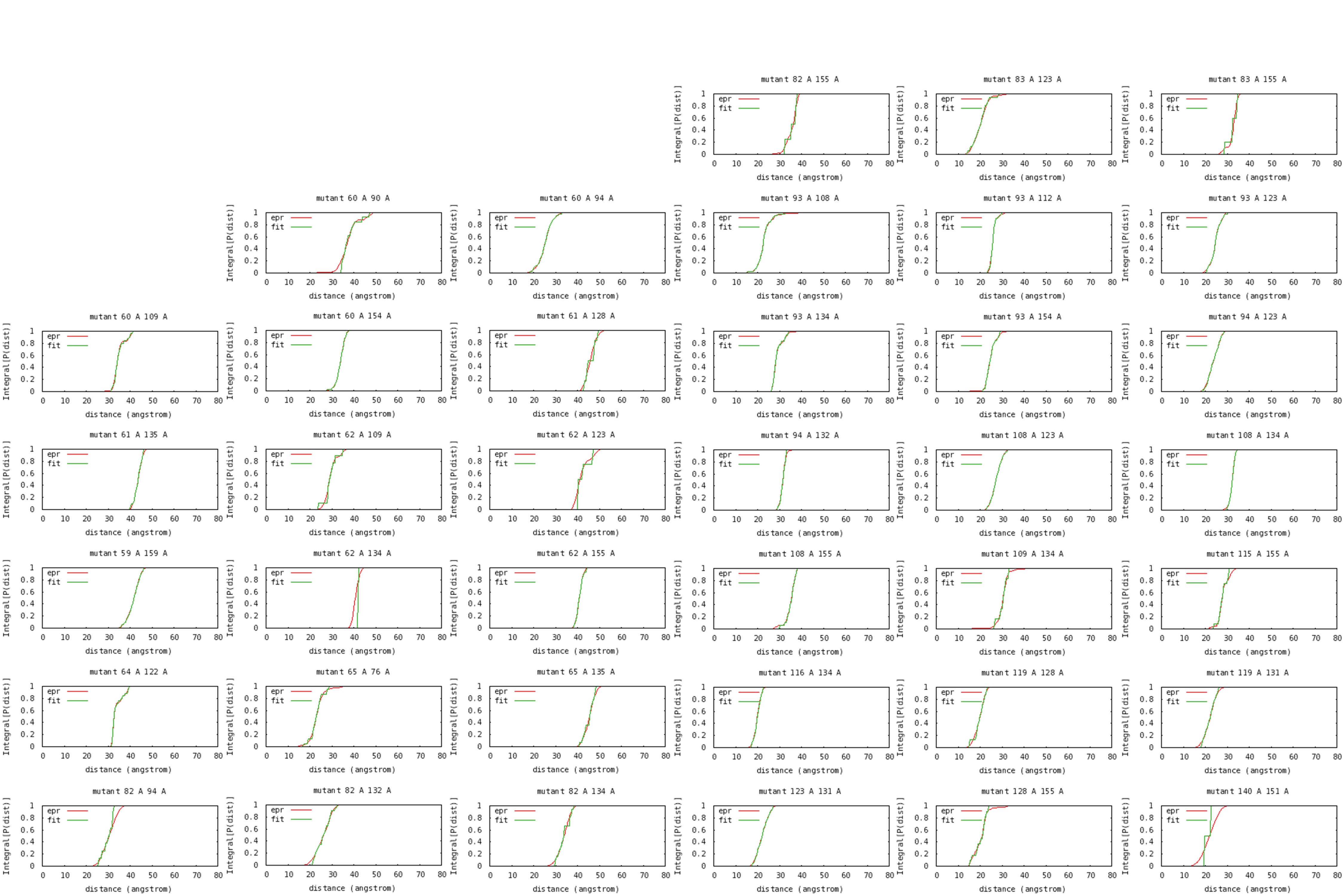

Supplement: Figure S6 — Agreement between experimental distance probability distributions and an ensemble of Rosetta models fitted to the experimental distribution for 38 double mutants of t4-lysozyme. Curves show the integral of the probability up to a given distance. (TIF) [file pone.0072851.s006.tif]

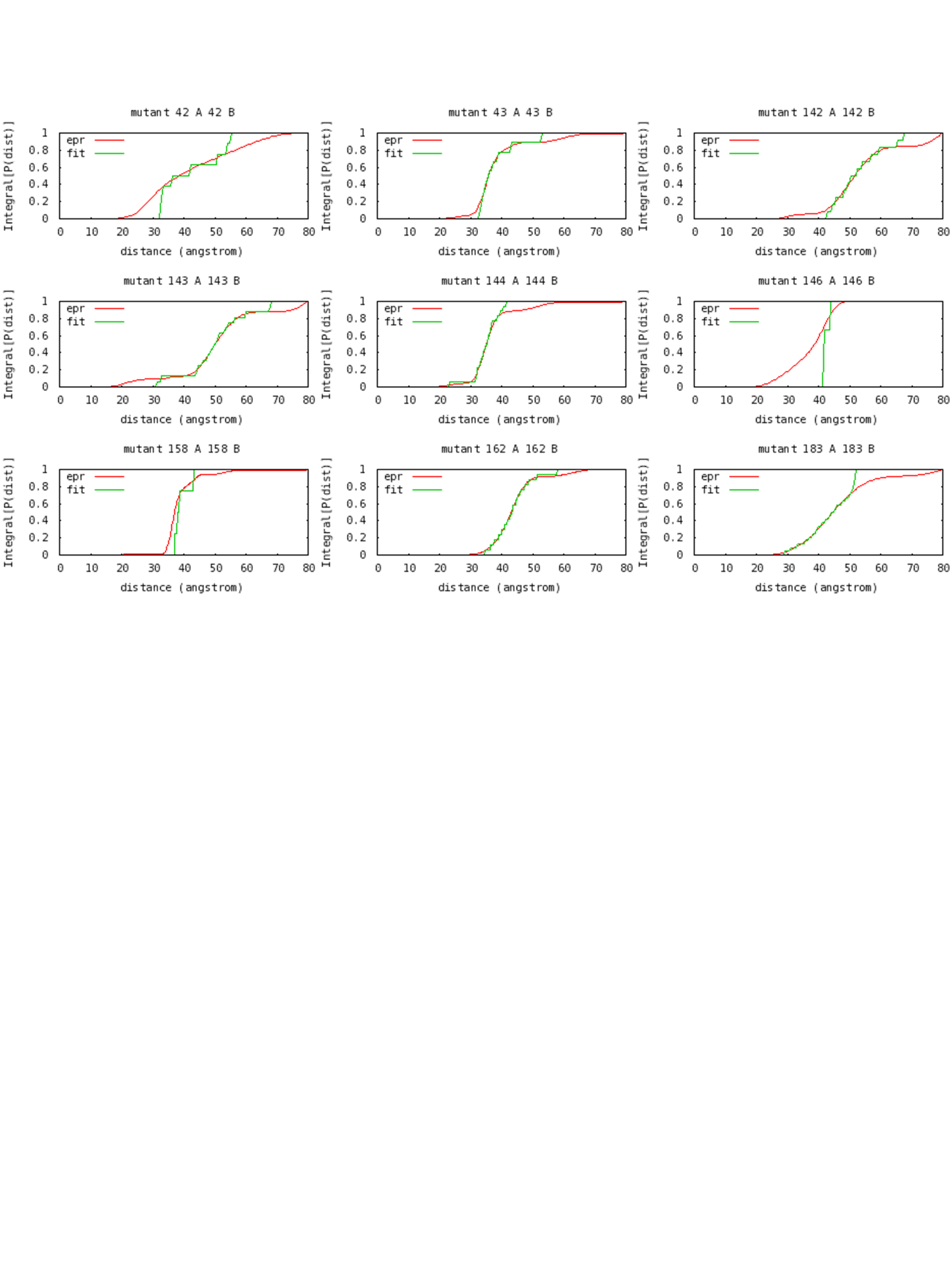

Supplement: Figure S7 — Agreement between experimental distance probability distributions and an ensemble of Rosetta models fitted to the experimental distribution for double mutants of MSBA in the apo-open state. Curves show the integral of the probability up to a given distance. (TIF) [file pone.0072851.s007.tif]

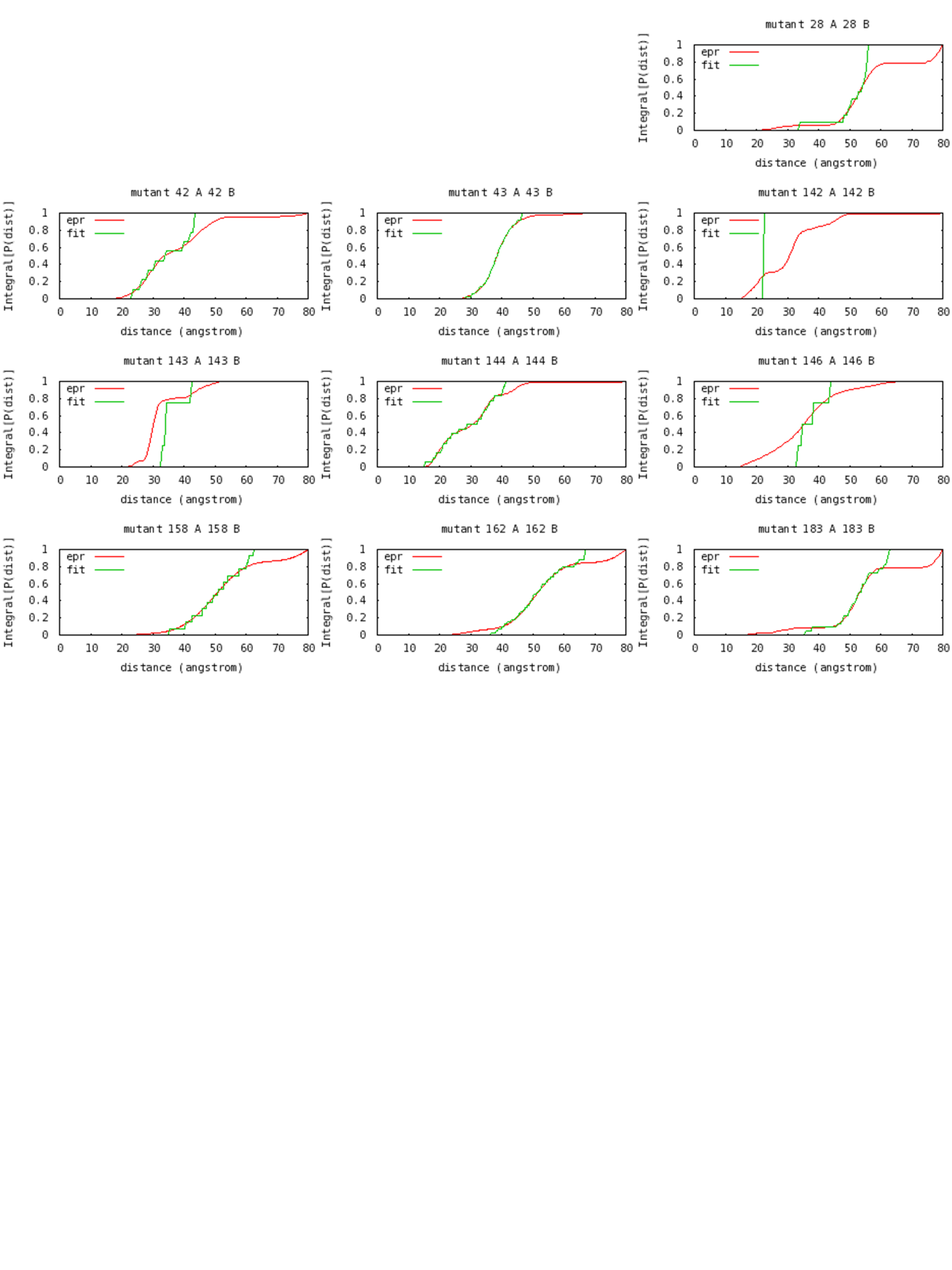

Supplement: Figure S8 — Agreement between experimental distance probability distributions and an ensemble of Rosetta models fitted to the experimental distribution for double mutants of MSBA in the AMP-PNP bound state. Curves show the integral of the probability up to a given distance. (TIF) [file pone.0072851.s008.tif]

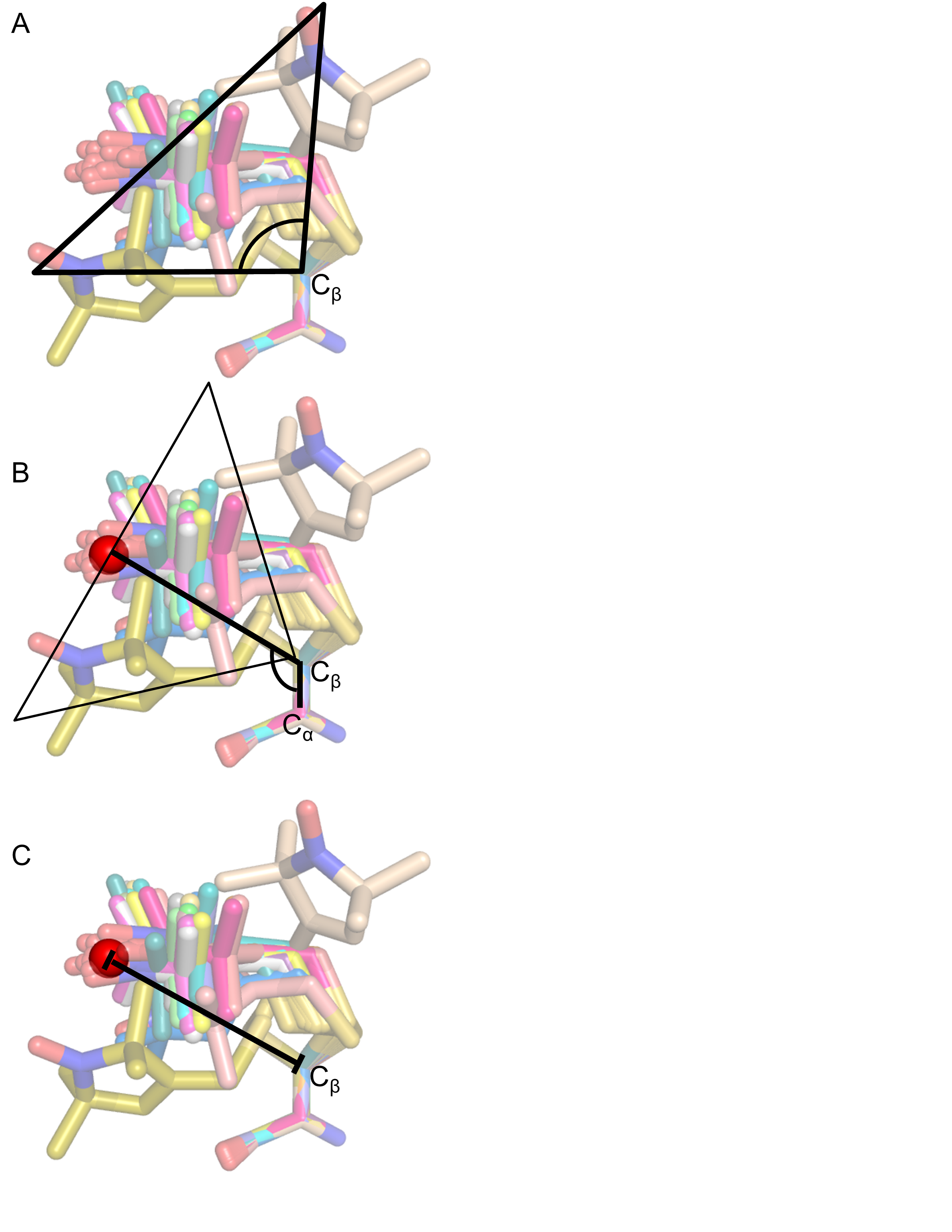

Supplement: Figure S9 — Visual description of the three parameters that define the cone model and their relation to the full-atom representation of the spin label. The effective spin label position, SLef, is the average position of the midpoint of the N-O bond vector. In B.) and C.) the SLef position is represented as a red sphere. A.) is the opening angle of the cone and is calculated as the widest angle observed between two MTSSL conformations obtained from Rosetta. B.) is the angle defined by the Cα, Cβ, and SLef positions, and gives information on the allowable tilt angles of the cone. C.) is the distance from the Cβ to the SLef position. (TIF) [file pone.0072851.s009.tif]

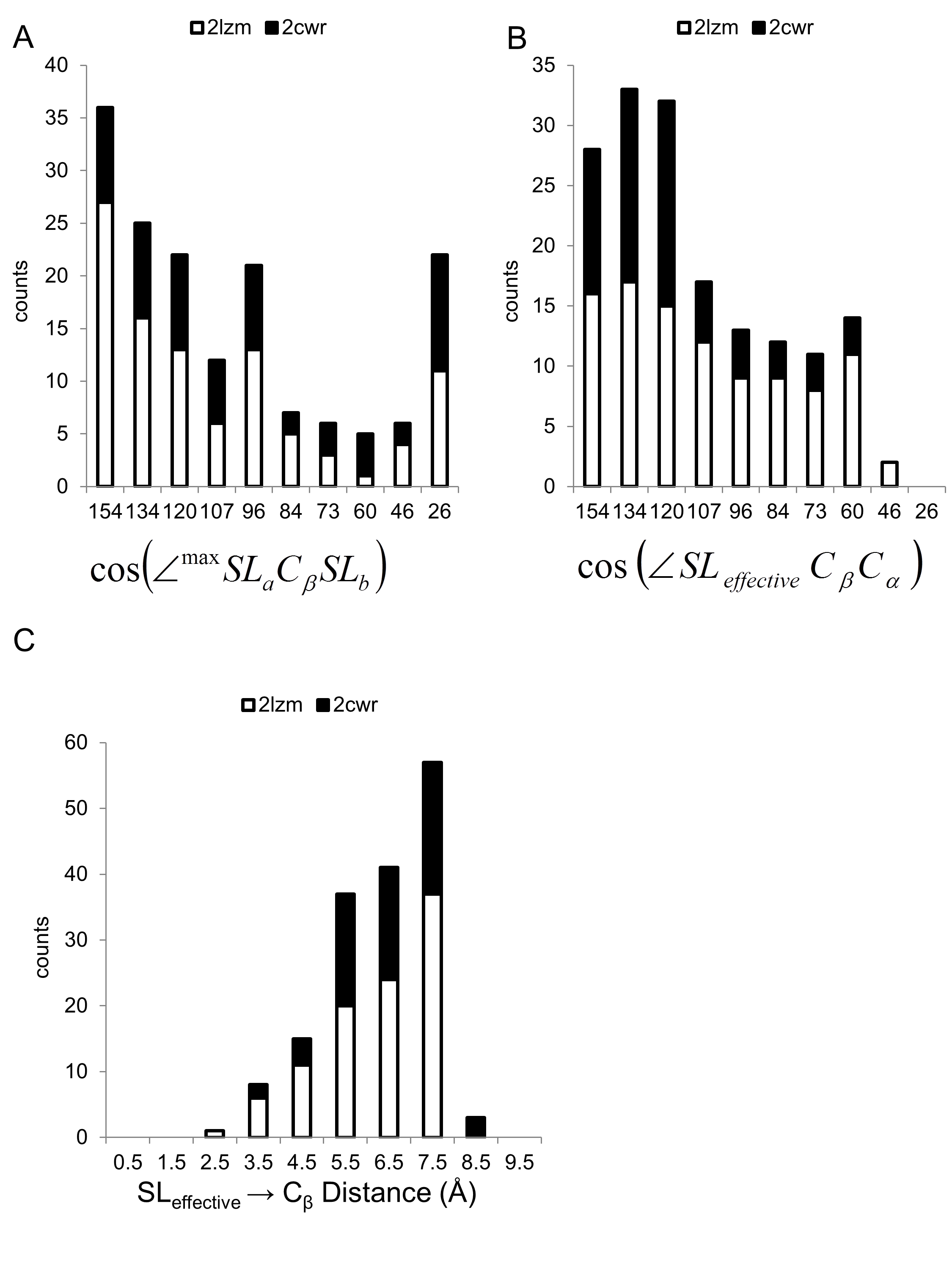

Supplement: Figure S10 — Distributions of the parameters that define the “cone model” as determined by Rosetta using the rotamer library full-atom representation of MTSSL. Shown are the frequencies with which given values of A.) B.) , and C.) are observed by Rosetta at 162 singly labeled MTSSL sites on primarily alpha-helical and beta-strand proteins. (TIF) [file pone.0072851.s010.tif]

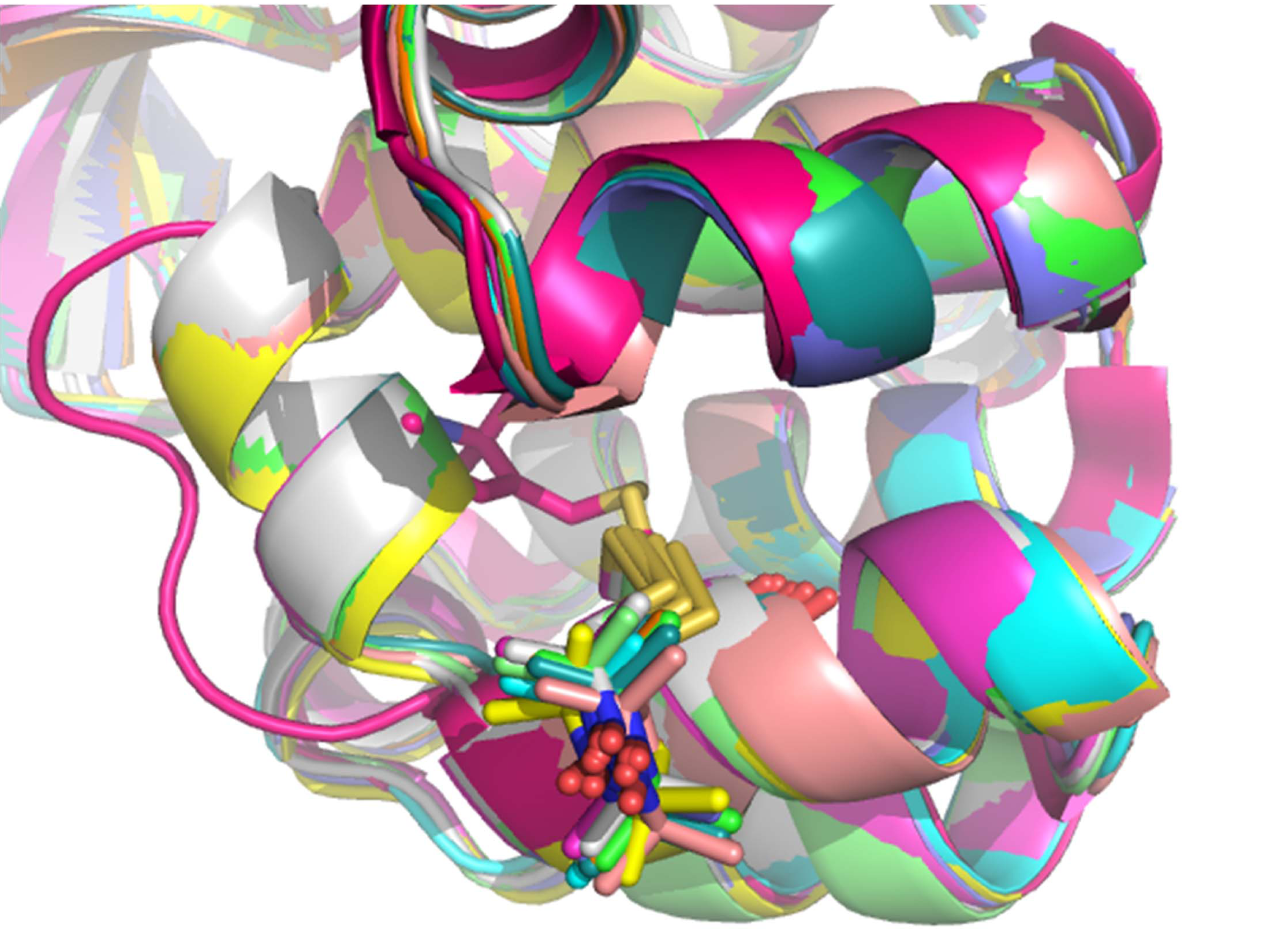

Supplement: Figure S11 — Relaxation of T4-lysozyme single mutant L118R1A starting from non-mutant crystal structure. The crystal structure of the T4-lysozyme single mutant L118R1A (PDB ID 2NTH) is shown in magenta. The pseudo-wildtype structure described in “Experimental Procedures” based on the crystal structure with PDB ID 2LZM was computationally mutated to contain a spin label at site 118 and relaxed ten times. The ten structures are shown. Residues 108–113 are unstructured in 2NTH, allowing space to accommodate the spin label. The corresponding helical residues in 2LZM remain structured after relaxation and the spin label is necessarily placed in an orientation different from that seen in 2NTH in order to avoid backbone clashes. (TIF) [file pone.0072851.s011.tif]

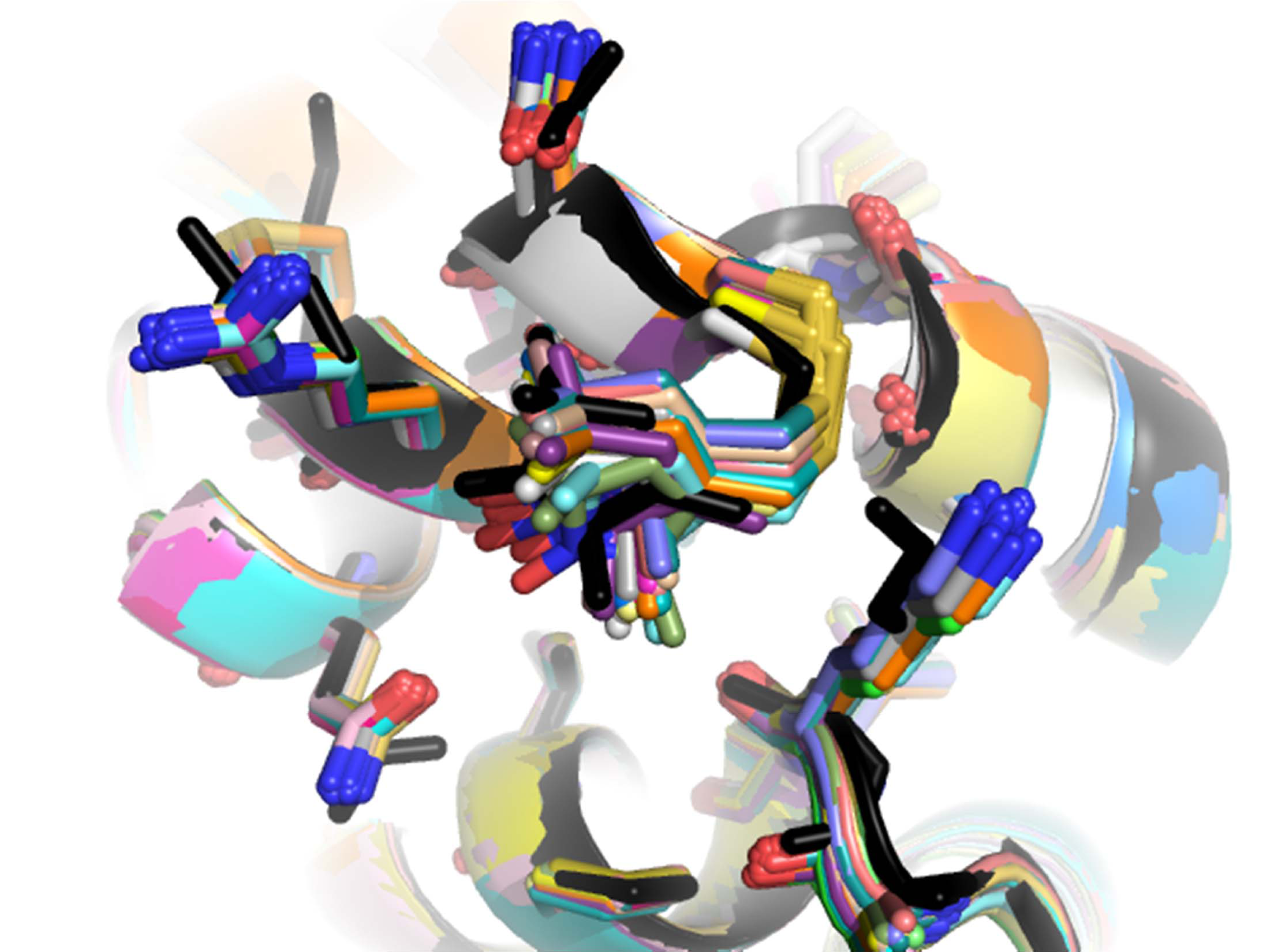

Supplement: Figure S12 — T4-lysozyme single mutant T115100R1A is the only non-crystal contact surface site where all five Χ angles have been observed. The structure has PDB ID identifier 2IGC and is shown in black. Out of the 1000 relaxation trajectories, twenty-four structures have the correct conformation of the spin label. The surrounding residues within 5 Å of the spin label are shown in sticks. (TIF) [file pone.0072851.s012.tif]

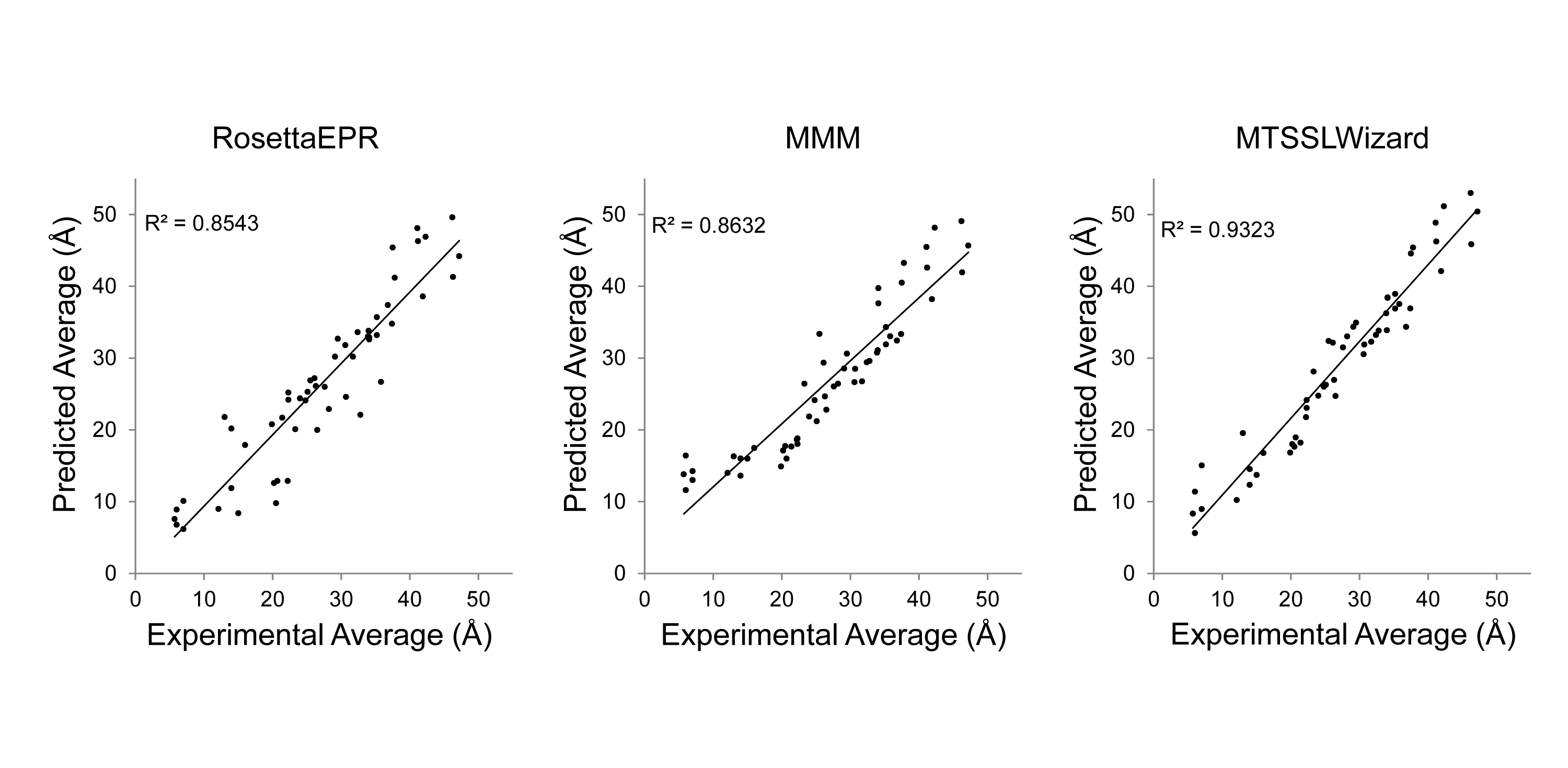

Supplement: Figure S13 — Plots of the average spin label distance from T4-lysozyme spin labeled double mutant distance distributions predicted by RosettaEPR, MMM, and MTSSLWizard compared to the experimental average distance measured by EPR. (TIF) [file pone.0072851.s013.tif]

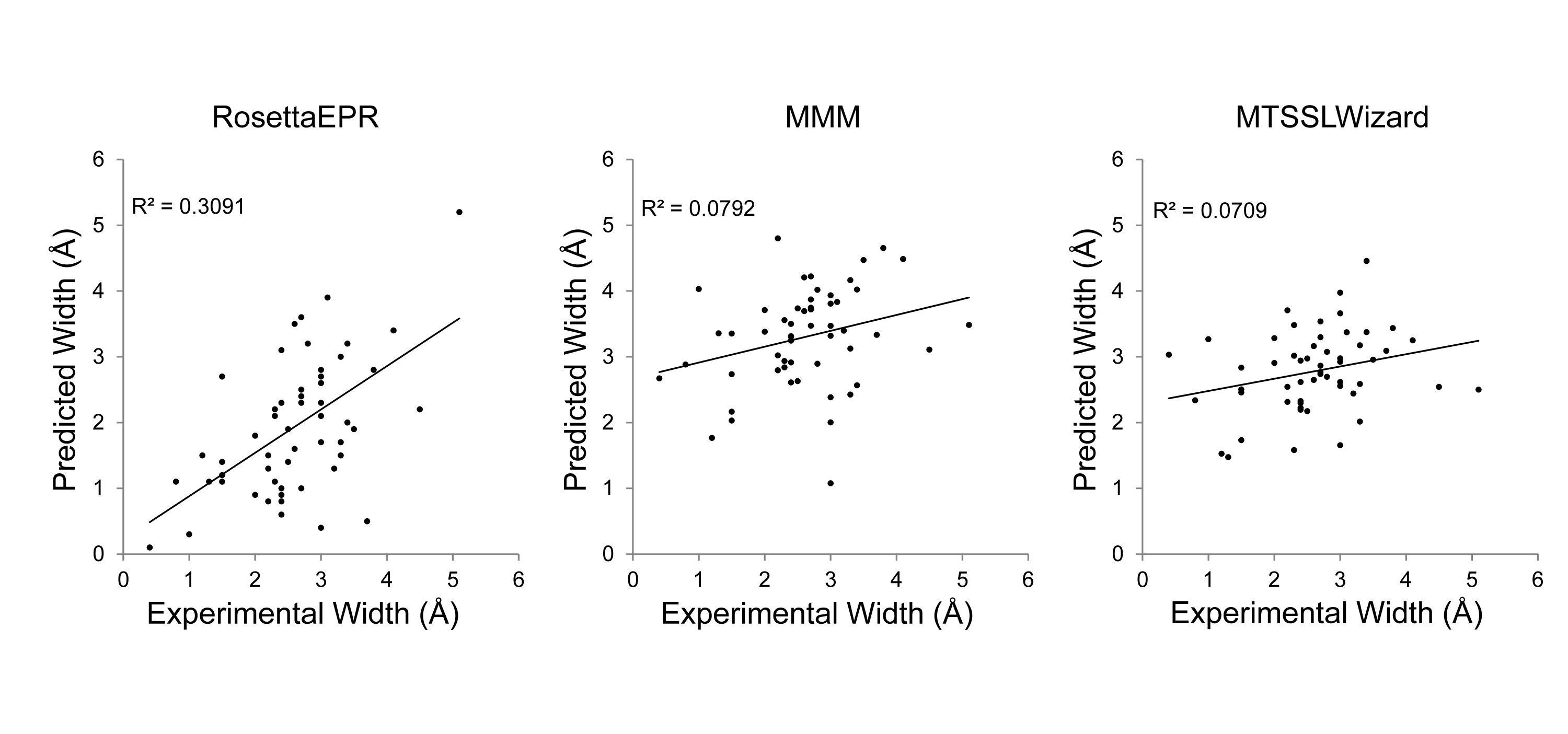

Supplement: Figure S14 — Plots of the standard deviation of spin labeled double mutant T4-lysozyme distance distributions predicted by RosettaEPR, MMM, and MTSSLWizard compared to the experimental standard deviation measured by EPR. (TIF) [file pone.0072851.s014.tif]

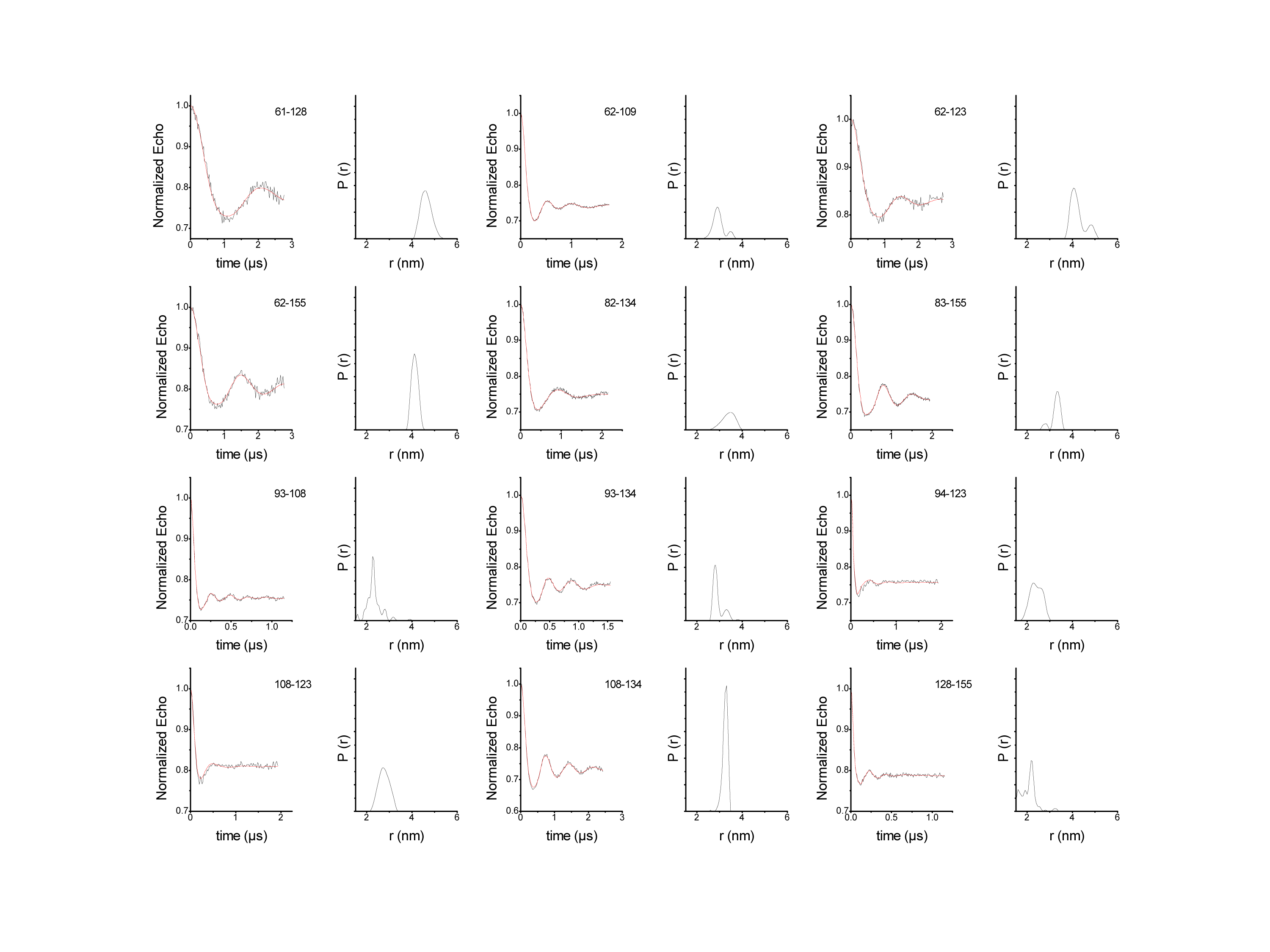

Supplement: Figure S15 — For spin labeled double mutants of T4-lysozyme, background-corrected normalized echo decay traces from DEER measurements with corresponding distance distributions obtained from Tikhonov regularization. (TIF) [file pone.0072851.s015.tif]
