# Supplementary material for: RosettaEPR: Rotamer Library for Spin Label Structure and Dynamics
Source: PLoS One. 2013 Sep 5;8(9):e72851. doi: 10.1371/journal.pone.0072851 (PMC3764097; doi:10.1371/journal.pone.0072851)
Supplement: Table S1 — Experimentally determined MTSSL conformations for single mutants of T4-lysozyme. (DOC) [file pone.0072851.s016.doc]

**Supplemental Table 1.** Experimentally determined MTSSL conformations for single mutants of T4-lysozyme.

| Mutant | Temp. (K) | Environ. | SSE Type | Rotamer | Χ_1_(°) | Χ_2_(°) | Χ_3_(°) | Χ_4_(°) | Χ_5_(°) | PDB ID | Ref. |
| --- | --- | --- | --- | --- | --- | --- | --- | --- | --- | --- | --- |
| R080 | 298 | surface | helix | {m,m} | -74 | -66 |  |  |  |  |  |
| R119 | 100 | surface | helix | {m,m} | -50 | -50 |  |  |  |  |  |
| R119 | 100 | surface | helix | {t,p} | 175 | 54 |  |  |  |  |  |
| K065 | 298 | crystal contact | helix | {t,p} | 153 | 89 | 53 |  |  |  |  |
| V075 | 100 | crystal contact | helix | {m,t} | -73 | 173 | 91 | 95 |  |  |  |
| T115 | 100 | surface | helix | {m,m} | -81 | -57 | -92 | 76 | 98 | 2IGC |  |
| T115/  R119A | 100 | surface | helix | {m,m} | -77 | -33 |  |  |  | 2OU9 |  |
| T115 | 298 | surface | helix | {m,m} | -94 | -28 |  |  |  | 2OU8 |  |
| T115 | 298 | surface | helix | {t,m} | 163 | -63 |  |  |  | 2OU8 |  |
| L118 | 100 | core | helix |  | -104 | 32 | 88 | 54 | 107 | 2NTH |  |
| A041 | 100 | crystal contact | helix | {t,p} | -175 | 57 | 86 |  |  | 2Q9D |  |
| S044_a_ | 100 | crystal contact | helix | {m,m} | -83 | -58 | -95 | 76 | -86 | 2Q9E |  |
| S044_b_ | 100 | crystal contact | helix | {m,m} | -85 | -55 | -96 | 71 | -78 | 2Q9E |  |
| S044_c_ | 100 | surface | helix | {t,m} | 173 | -96 |  |  |  | 2Q9E |  |
| A082 | 100 | surface | loop | {m,m} | -68 | -56 | 101 |  |  | 1ZYT |  |
| V131 | 100 | surface | helix | {m,m} | -69 | -60 |  |  |  | 2CUU |  |
| V131 | 100 | surface | helix | {t,p} | 175 | 80 |  |  |  | 2CUU |  |
| V131 | 291 | surface | helix | {m,m} | -75 | -57 |  |  |  | 3G3V |  |
| V131 | 291 | surface | helix | {t,p} | 175 | 83 |  |  |  | 3G3V |  |
| T151 | 100 | surface | helix | {m,m} | -83 | -72 |  |  |  | 3G3X |  |
| T151 | 291 | surface | helix | {m,m} | -82 | -72 |  |  |  | 3G3W |  |

*Mutant* indicates the residue of t4-lysozyme which was mutated to the MTSSL side chain. Subscripts denote the protein subunit from the crystal structure asymmetric unit as indicated in the PDB file. *Temp.* gives the temperature at which the crystal was formed. *Environ.* gives the environment in which the residue lies: on the surface of the protein (surface); within the core of the protein (core); at the contact point of two a crystallographic subunits (crystal contact). *SSE Type* gives the type of secondary structure element on which the mutated residue sits. *Rotamer* indicates the Χ_1_ and Χ_2_ angles observed for the spin label in the crystal structure according to the m, t, p convention of . The Χ angles observed in the crystal structure are shown in their respective columns. Blank columns indicate the Χ angles were not resolved. *PDB ID* is the Protein Data Bank accession identifier of the crystal structure, if available. *Ref* provides the primary citation for the crystal structure.

1. Langen R, Oh KJ, Cascio D, Hubbell WL (2000) Crystal structures of spin labeled T4 lysozyme mutants: Implications for the interpretation of EPR spectra in terms of structure. Biochemistry 39: 8396-8405.

2. Lovell SC, Word JM, Richardson JS, Richardson DC (2000) The penultimate rotamer library. Proteins-Structure Function and Genetics 40: 389-408.

3. Guo ZF, Cascio D, Hideg K, Kalai T, Hubbell WL (2007) Structural determinants of nitroxide motion in spin-labeled proteins: Tertiary contact and solvent-inaccessible sites in helix G of T4 lysozyme. Protein Science 16: 1069-1086.

4. Guo ZF, Cascio D, Hideg K, Hubbell WL (2008) Structural determinants of nitroxide motion in spin-labeled proteins: Solvent-exposed sites in helix B of T4 lysozyme. Protein Science 17: 228-239.

5. Fleissner MR, Cascio D, Hubbell WL (2009) Structural origin of weakly ordered nitroxide motion in spin-labeled proteins. Protein Science 18: 893-908.
