# Supplementary material for: RosettaEPR: Rotamer Library for Spin Label Structure and Dynamics
Source: PLoS One. 2013 Sep 5;8(9):e72851. doi: 10.1371/journal.pone.0072851 (PMC3764097; doi:10.1371/journal.pone.0072851)
Supplement: Table S2 — Experimentally determined MTSSL conformations for single mutants of LeuT. (DOC) [file pone.0072851.s017.doc]

**Supplemental Table 1**. Experimentally determined MTSSL conformations for single mutants of LeuT.

| Mutant | Temp. (K) | Environ. | SSE Type | Rotamer | Χ_1_(°) | Χ_2_(°) | Χ_3_(°) | Χ_4_(°) | Χ_5_(°) | PDB ID | Ref. |
| --- | --- | --- | --- | --- | --- | --- | --- | --- | --- | --- | --- |
| F177 | 100 | Surface | Helix | {m,m} | -69 | -57 | 107 | 103 | -24 | 3MPN |  |
| I204 | 100 | Surface | Helix | {m,m} | -69 | -59 | -87 | -71 | -95 | 3MPQ |  |

*Mutant* indicates the residue of LeuT which was mutated to the MTSSL side chain. Subscripts denote the protein subunit from the crystal structure asymmetric unit as indicated in the PDB file. *Temp.* gives the temperature at which the crystal was formed. *Environ.* gives the environment in which the residue lies: on the surface of the protein (surface); within the core of the protein (core); at the contact point of two a crystallographic subunits (crystal contact). *SSE Type* gives the type of secondary structure element on which the mutated residue sits. *Rotamer* indicates the Χ_1_ and Χ_2_ angles observed for the spin label in the crystal structure according to the m, t, p convention of . The Χ angles observed in the crystal structure are shown in their respective columns. *PDB ID* is the Protein Data Bank accession identifier of the crystal structure. *Ref* provides the primary citation for the crystal structure.

1. Kroncke BM, Horanyi PS, Columbus L (2010) Structural Origins of Nitroxide Side Chain Dynamics on Membrane Protein Î±-Helical Sites. Biochemistry 49: 10045-10060.

2. Lovell SC, Word JM, Richardson JS, Richardson DC (2000) The penultimate rotamer library. Proteins-Structure Function and Genetics 40: 389-408.
