# Supplementary material for: RosettaEPR: Rotamer Library for Spin Label Structure and Dynamics
Source: PLoS One. 2013 Sep 5;8(9):e72851. doi: 10.1371/journal.pone.0072851 (PMC3764097; doi:10.1371/journal.pone.0072851)
Supplement: Table S3 — Combinations of Χ1 and Χ2 leading to the combinations contained in the rotamer library. (DOC) [file pone.0072851.s018.doc]

**Supplemental Table 1**. Combinations of Χ_1_ and Χ_2_ leading to the combinations contained in the rotamer library.

| {m,m} | | {t,p} | | {t,m} | | {m,t} | | {t,t} | |
| --- | --- | --- | --- | --- | --- | --- | --- | --- | --- |
| -68 | -56 | 175 | 80 | 166 | -58 | -73 | 173 | 180 | 180 |
| -69 | -60 | 175 | 83 | 173 | -96 |  | | | |
| -81 | -57 | 175 | 54 |  | |
| -88 | -29 | 153 | 89 |
| -78 | -33 | 185 | 57 |
| -83 | -57 |  | |
| -85 | -55 |
| -75 | -57 |
| -82 | -72 |
| -83 | -72 |
| -50 | -50 |
| -74 | -66 |
| -76 | -56 | 173 | 73 | 170 | -77 | -73 | 173 | 180 | 180 |
| 10 | 13 | 11 | 14 | 3 | 19 | 0 | 0 | 0 | 0 |

Only the {t,t} combination is not observed experimentally and solely predicted from computational methods. The bottom two rows show the average and standard deviation, respectively.
