# Supplementary material for: RosettaEPR: Rotamer Library for Spin Label Structure and Dynamics
Source: PLoS One. 2013 Sep 5;8(9):e72851. doi: 10.1371/journal.pone.0072851 (PMC3764097; doi:10.1371/journal.pone.0072851)
Supplement: Table S4 — The average (μ) and standard deviation (σ) of inter-spin label distance distributions for double mutants of T4 lysozyme. (DOC) [file pone.0072851.s019.doc]

**Supplemental Table 1.** The average (μ) and standard deviation (σ) of inter-spin label distance distributions for double mutants of T4 lysozyme.

| AA_1_ | AA_2_ | μ _Rosetta_ | σ _Rrosetta_ | μ _EPR_ | σ _EPR_ | |μ _Rosetta_ - μ _EPR_| | |σ _Rrosetta_ - σ _EPR_| |
| --- | --- | --- | --- | --- | --- | --- | --- |
| 59 | 159 | 38.6 | 3.6 | 41.9 | 2.7 | 3.3 | 0.9 |
| 60 | 90 | 41.2 | 2.2 | 37.8 | 4.5 | 3.4 | 2.3 |
| 60 | 94 | 26.9 | 3.9 | 25.5 | 3.1 | 1.4 | 0.8 |
| 60 | 109 | 35.7 | 1.6 | 35.2 | 2.6 | 0.5 | 1.0 |
| 60 | 154 | 32.6 | 1.8 | 34.1 | 2.0 | 1.5 | 0.2 |
| 61 | 80 | 33.8 | 1.3 | 34.0 | 2.2 | 0.2 | 0.9 |
| 61 | 86 | 45.4 | 0.9 | 37.5 | 2.0 | 7.9 | 1.1 |
| 61 | 128 | 49.6 | 0.6 | 46.2 | 2.4 | 3.4 | 1.8 |
| 61 | 135 | 44.2 | 0.8 | 47.2 | 2.2 | 3.0 | 1.4 |
| 62 | 109 | 32.7 | 2.5 | 29.5 | 2.7 | 3.2 | 0.2 |
| 62 | 123 | 46.9 | 1.7 | 42.3 | 3.3 | 4.6 | 1.6 |
| 62 | 134 | 48.1 | 1.1 | 41.1 | 1.5 | 7.0 | 0.4 |
| 62 | 155 | 46.3 | 1.2 | 41.2 | 1.5 | 5.1 | 0.3 |
| 64 | 122 | 32.9 | 1.4 | 34.1 | 2.5 | 1.2 | 1.1 |
| 65 | 76 | 21.7 | 3.2 | 21.4 | 2.8 | 0.3 | 0.4 |
| 65 | 80 | 20.0 | 2.8 | 26.5 | 3.8 | 6.5 | 1.0 |
| 65 | 86 | 34.8 | 2.3 | 37.4 | 2.7 | 2.6 | 0.4 |
| 65 | 135 | 41.3 | 1.5 | 46.3 | 2.2 | 5.0 | 0.7 |
| 80 | 135 | 37.4 | 0.3 | 36.8 | 1.0 | 0.6 | 0.7 |
| 82 | 94 | 24.6 | 1.5 | 30.7 | 3.3 | 6.1 | 1.8 |
| 82 | 132 | 26.1 | 1.9 | 26.3 | 3.5 | 0.2 | 1.6 |
| 82 | 134 | 33.0 | 1.3 | 33.9 | 3.2 | 0.9 | 1.9 |
| 82 | 155 | 26.7 | 1.9 | 35.8 | 2.5 | 9.1 | 0.6 |
| 83 | 123 | 9.8 | 2.0 | 20.5 | 3.4 | 10.7 | 1.4 |
| 83 | 155 | 22.1 | 2.1 | 32.8 | 3.0 | 10.7 | 0.9 |
| 86 | 112 | 21.8 | 5.2 | 13.0 | 5.1 | 8.8 | 0.1 |
| 86 | 119 | 8.4 | 2.7 | 15.0 | 3.0 | 6.6 | 0.3 |
| 88 | 100 | 8.9 | 1.7 | 6.0 | 3.0 | 2.9 | 1.3 |
| 89 | 93 | 17.9 | 2.6 | 16.0 | 3.0 | 1.9 | 0.4 |
| 89 | 96 | 6.8 | 2.8 | 6.0 | 3.0 | 0.8 | 0.2 |
| 93 | 108 | 20.1 | 3.4 | 23.3 | 4.1 | 3.2 | 0.7 |
| 93 | 112 | 27.2 | 1.4 | 26.1 | 1.5 | 1.1 | 0.1 |
| 93 | 123 | 24.1 | 2.2 | 24.8 | 2.3 | 0.7 | 0.1 |
| 93 | 134 | 30.2 | 0.8 | 29.1 | 2.4 | 1.1 | 1.6 |
| 93 | 154 | 25.3 | 0.9 | 25.1 | 2.4 | 0.2 | 1.5 |
| 94 | 123 | 24.4 | 3.5 | 24.0 | 2.6 | 0.4 | 0.9 |
| 94 | 132 | 30.2 | 1.1 | 31.7 | 1.3 | 1.5 | 0.2 |
| 108 | 123 | 26.0 | 3.1 | 27.6 | 2.4 | 1.6 | 0.7 |
| 108 | 134 | 33.6 | 1.5 | 32.4 | 1.2 | 1.2 | 0.3 |
| 108 | 155 | 33.2 | 1.1 | 35.2 | 2.3 | 2.0 | 1.2 |
| 109 | 134 | 31.8 | 3.2 | 30.6 | 2.8 | 1.2 | 0.4 |
| 115 | 155 | 22.9 | 1.0 | 28.2 | 2.4 | 5.3 | 1.4 |
| 116 | 134 | 12.6 | 2.7 | 20.2 | 1.5 | 7.6 | 1.2 |
| 119 | 128 | 20.8 | 2.1 | 19.9 | 2.3 | 0.9 | 0.2 |
| 119 | 131 | 24.2 | 1.0 | 22.3 | 2.7 | 1.9 | 1.7 |
| 120 | 131 | 20.2 | 0.4 | 14.0 | 3.0 | 6.2 | 2.6 |
| 123 | 131 | 25.2 | 2.4 | 22.3 | 2.7 | 2.9 | 0.3 |
| 127 | 151 | 11.9 | 2.3 | 14.0 | 2.4 | 2.1 | 0.1 |
| 127 | 154 | 10.1 | 2.3 | 7.0 | 3.0 | 3.1 | 0.7 |
| 127 | 155 | 9.0 | 3.2 | 12.1 | 3.4 | 3.1 | 0.2 |
| 128 | 155 | 12.9 | 0.5 | 20.7 | 3.7 | 7.8 | 3.2 |
| 131 | 150 | 7.6 | 0.1 | 5.7 | 0.4 | 1.9 | 0.3 |
| 134 | 151 | 6.2 | 1.1 | 7.0 | 0.8 | 0.8 | 0.3 |
| 140 | 151 | 12.9 | 3.0 | 22.2 | 3.3 | 9.3 | 0.3 |
| μ |  | | | | | 3.5 | 0.9 |
| σ |  | | | | | 2.9 | 0.7 |
| RMSD |  | | | | | 4.5 | 1.1 |
| R |  | | | | | 0.92 | 0.56 |

AA_1_ and AA_2_ denote the sites of mutation. Values are calculated from the best 200 Rosetta models according to score and from EPR experiment, respectively. The deviation of Rosetta from experiment in average (μ) and standard deviation (σ) is also given for each double mutant. The bottom four rows show the mean deviation, standard deviation of the deviation, RMSD, and the correlation coefficient (R) of Rosetta with experiment. Double mutants 116-131, 131-151, 131-154, and 140-147 are not included in the statistics calculations.
