# Supplementary material for: RosettaEPR: Rotamer Library for Spin Label Structure and Dynamics
Source: PLoS One. 2013 Sep 5;8(9):e72851. doi: 10.1371/journal.pone.0072851 (PMC3764097; doi:10.1371/journal.pone.0072851)
Supplement: Table S7 — Using Cβ atoms to approximate the position of spin labels in T4 lysozyme. (DOC) [file pone.0072851.s022.doc]

**Supplemental Table 1.** Using Cβ atoms to approximate the position of spin labels in T4 lysozyme.

| AA_1_ | AA_2_ | $\mu_{Rosetta}^{C\beta}$ | $\sigma_{Rosetta}^{C\beta}$ | μ _EPR_ | σ _EPR_ | $\left\vert\mu_{Rosetta}^{C\beta}-\mu_{EPR}\right\vert$ | $\left\vert\sigma_{Rosetta}^{C\beta}-\sigma_{EPR}\right\vert$ |
| --- | --- | --- | --- | --- | --- | --- | --- |
| 59 | 159 | 33.5 | 0.2 | 41.9 | 2.7 | 8.4 | 2.5 |
| 60 | 90 | 36.6 | 0.2 | 37.8 | 4.5 | 1.2 | 4.3 |
| 60 | 94 | 28.1 | 0.6 | 25.5 | 3.1 | 2.6 | 2.5 |
| 60 | 109 | 31.0 | 0.3 | 35.2 | 2.6 | 4.2 | 2.3 |
| 60 | 154 | 34.1 | 0.4 | 34.1 | 2.0 | 0.0 | 1.6 |
| 61 | 80 | 28.3 | 0.1 | 34.0 | 2.2 | 5.7 | 2.1 |
| 61 | 86 | 36.5 | 0.1 | 37.5 | 2.0 | 1.0 | 1.9 |
| 61 | 128 | 42.6 | 0.3 | 46.2 | 2.4 | 3.6 | 2.1 |
| 61 | 135 | 39.9 | 0.3 | 47.2 | 2.2 | 7.3 | 1.9 |
| 62 | 109 | 27.0 | 0.3 | 29.5 | 2.7 | 2.5 | 2.4 |
| 62 | 123 | 41.3 | 0.2 | 42.3 | 3.3 | 1.0 | 3.1 |
| 62 | 134 | 35.5 | 0.3 | 41.1 | 1.5 | 5.6 | 1.2 |
| 62 | 155 | 34.8 | 0.2 | 41.2 | 1.5 | 6.4 | 1.3 |
| 64 | 122 | 33.6 | 0.2 | 34.1 | 2.5 | 0.5 | 2.3 |
| 65 | 76 | 16.5 | 0.1 | 21.4 | 2.8 | 4.9 | 2.7 |
| 65 | 80 | 22.1 | 0.1 | 26.5 | 3.8 | 4.4 | 3.7 |
| 65 | 86 | 30.9 | 0.1 | 37.4 | 2.7 | 6.5 | 2.6 |
| 65 | 135 | 36.1 | 0.2 | 46.3 | 2.2 | 10.2 | 2.0 |
| 80 | 135 | 26.6 | 0.2 | 36.8 | 1.0 | 10.2 | 0.8 |
| 82 | 94 | 23.0 | 0.1 | 30.7 | 3.3 | 7.7 | 3.2 |
| 82 | 132 | 19.9 | 0.2 | 26.3 | 3.5 | 6.4 | 3.3 |
| 82 | 134 | 25.3 | 0.2 | 33.9 | 3.2 | 8.6 | 3.0 |
| 82 | 155 | 27.5 | 0.1 | 35.8 | 2.5 | 8.3 | 2.4 |
| 83 | 123 | 14.2 | 0.3 | 20.5 | 3.4 | 6.3 | 3.1 |
| 83 | 155 | 23.9 | 0.2 | 32.8 | 3.0 | 8.9 | 2.8 |
| 86 | 112 | 11.2 | 0.3 | 13.0 | 5.1 | 1.8 | 4.8 |
| 86 | 119 | 10.3 | 0.3 | 15.0 | 3.0 | 4.7 | 2.7 |
| 88 | 100 | 8.3 | 0.3 | 6.0 | 3.0 | 2.3 | 2.7 |
| 89 | 93 | 12.1 | 0.1 | 16.0 | 3.0 | 3.9 | 2.9 |
| 89 | 96 | 8.6 | 0.2 | 6.0 | 3.0 | 2.6 | 2.8 |
| 93 | 108 | 20.9 | 0.4 | 23.3 | 4.1 | 2.4 | 3.7 |
| 93 | 112 | 21.7 | 0.2 | 26.1 | 1.5 | 4.4 | 1.3 |
| 93 | 123 | 18.5 | 0.1 | 24.8 | 2.3 | 6.3 | 2.2 |
| 93 | 134 | 23.0 | 0.2 | 29.1 | 2.4 | 6.1 | 2.2 |
| 93 | 154 | 15.6 | 0.2 | 25.1 | 2.4 | 9.5 | 2.2 |
| 94 | 123 | 17.4 | 0.2 | 24.0 | 2.6 | 6.6 | 2.4 |
| 94 | 132 | 18.2 | 0.2 | 31.7 | 1.3 | 13.5 | 1.1 |
| 108 | 123 | 21.3 | 0.2 | 27.6 | 2.4 | 6.3 | 2.2 |
| 108 | 134 | 21.4 | 0.2 | 32.4 | 1.2 | 11.0 | 1.0 |
| 108 | 155 | 25.2 | 0.3 | 35.2 | 2.3 | 10.0 | 2.0 |
| 109 | 134 | 20.4 | 0.2 | 30.6 | 2.8 | 10.2 | 2.6 |
| 115 | 155 | 22.5 | 0.2 | 28.2 | 2.4 | 5.7 | 2.2 |
| 116 | 134 | 11.3 | 0.3 | 20.2 | 1.5 | 8.9 | 1.2 |
| 119 | 128 | 8.7 | 0.2 | 19.9 | 2.3 | 11.2 | 2.1 |
| 119 | 131 | 12.0 | 0.2 | 22.3 | 2.7 | 10.3 | 2.5 |
| 120 | 131 | 8.6 | 0.1 | 14.0 | 3.0 | 5.4 | 2.9 |
| 123 | 131 | 13.2 | 0.1 | 22.3 | 2.7 | 9.1 | 2.6 |
| 127 | 151 | 11.6 | 0.3 | 14.0 | 2.4 | 2.4 | 2.1 |
| 127 | 154 | 6.8 | 0.3 | 7.0 | 3.0 | 0.2 | 2.7 |
| 127 | 155 | 10.3 | 0.4 | 12.1 | 3.4 | 1.8 | 3.0 |
| 128 | 155 | 13.9 | 0.2 | 20.7 | 3.7 | 6.8 | 3.5 |
| 131 | 150 | 9.1 | 0.1 | 5.7 | 0.4 | 3.4 | 0.3 |
| 134 | 151 | 10.5 | 0.3 | 7.0 | 0.8 | 3.5 | 0.5 |
| 140 | 151 | 16.6 | 0.1 | 22.2 | 3.3 | 5.6 | 3.2 |
| μ |  | | | | | 5.7 | 2.4 |
| σ |  | | | | | 3.3 | 0.9 |
| RMSD |  | | | | | 6.6 | 2.5 |
| R |  | | | | | 0.93 | 0.10 |

Values are average (μ) and standard deviation (σ) of inter-Cβ distance distributions for double mutants (AA_1_ and AA_2_) of T4 lysozyme as calculated from the best 200 Rosetta models according to score and inter-spin label distance distributions from EPR experiment, respectively. The deviation of Rosetta from experiment in terms μ and σ is also given for each double mutant. The bottom four rows show the mean deviation, standard deviation of the deviation, RMSD, and the correlation coefficient (R) of Rosetta with experiment.
