# Supplementary material for: RosettaEPR: Rotamer Library for Spin Label Structure and Dynamics
Source: PLoS One. 2013 Sep 5;8(9):e72851. doi: 10.1371/journal.pone.0072851 (PMC3764097; doi:10.1371/journal.pone.0072851)
Supplement: Table S8 — Using Cβ atoms to approximate the position of spin labels in MSBA in the apo open state. (DOC) [file pone.0072851.s023.doc]

**Supplemental Table 1.** Using Cβ atoms to approximate the position of spin labels in MSBA in the apo open state.

| AA_1_ | AA_2_ | $\mu_{Rosetta}^{C\beta}$ | $\sigma_{Rosetta}^{C\beta}$ | μ _EPR_ | σ _EPR_ | $\left\vert\mu_{Rosetta}^{C\beta}-\mu_{EPR}\right\vert$ | $\left\vert\sigma_{Rosetta}^{C\beta}-\sigma_{EPR}\right\vert$ |
| --- | --- | --- | --- | --- | --- | --- | --- |
| 42 | 42 | 31.8 | 2.4 | 36 | 10 | 4.2 | 7.6 |
| 43 | 43 | 33.4 | 1.8 | 35 | 2.5 | 1.6 | 0.7 |
| 142 | 142 | 40.3 | 4.3 | 54 | 7 | 13.7 | 2.7 |
| 143 | 143 | 37.8 | 3.3 | 54 | 5.6 | 16.2 | 2.3 |
| 144 | 144 | 29.1 | 3.4 | 35 | 2.5 | 5.9 | 0.9 |
| 146 | 146 | 40.4 | 3.2 | 42 | 3.8 | 1.6 | 0.6 |
| 158 | 158 | 36.9 | 1.9 | 36 | 1.5 | 0.9 | 0.4 |
| 162 | 162 | 37.1 | 1.3 | 44 | 3.2 | 6.9 | 1.9 |
| 183 | 183 | 33.2 | 2.3 | 43 | 13.2 | 9.8 | 10.9 |
| μ |  | | | | | 6.8 | 3.1 |
| σ |  | | | | | 5.2 | 3.5 |
| RMSD |  | | | | | 8.5 | 4.6 |
| R |  | | | | | 0.67 | 0.14 |

Values are the average (μ) and standard deviation (σ) of inter-Cβ distance distributions for double mutants (AA_1_ and AA_2_) of MSBA in the apo open state as calculated from the best 100 Rosetta models according to score and inter-spin label distance distributions from EPR experiment, respectively. The deviation of Rosetta from experiment in terms μ and σ is also given for each double mutant. The bottom four rows show the mean deviation, standard deviation of the deviation, RMSD, and the correlation coefficient (R) of Rosetta with experiment.
