# Supplementary material for: RosettaEPR: Rotamer Library for Spin Label Structure and Dynamics
Source: PLoS One. 2013 Sep 5;8(9):e72851. doi: 10.1371/journal.pone.0072851 (PMC3764097; doi:10.1371/journal.pone.0072851)
Supplement: Table S9 — Using Cβ atoms to approximate the position of spin labels in MSBA in the AMP-PNP bound state. (DOC) [file pone.0072851.s024.doc]

**Supplemental Table 1.** Using Cβ atoms to approximate the position of spin labels in MSBA in the AMP-PNP bound state.

| AA_1_ | AA_2_ | $\mu_{Rosetta}^{C\beta}$ | $\sigma_{Rosetta}^{C\beta}$ | μ _EPR_ | σ _EPR_ | $\left\vert\mu_{Rosetta}^{C\beta}-\mu_{EPR}\right\vert$ | $\left\vert\sigma_{Rosetta}^{C\beta}-\sigma_{EPR}\right\vert$ |
| --- | --- | --- | --- | --- | --- | --- | --- |
| 28 | 28 | 40.9 | 0.7 | 53 | 4.2 | 12.1 | 3.5 |
| 42 | 42 | 24.4 | 2.4 | 36 | 12 | 11.6 | 9.6 |
| 43 | 43 | 29.4 | 2.5 | 38 | 3 | 8.6 | 0.5 |
| 142 | 142 | 18.7 | 0.9 | 30 | 7.5 | 11.3 | 6.6 |
| 143 | 143 | 26.5 | 0.8 | 26 | 1.5 | 0.5 | 0.7 |
| 144 | 144 | 21.5 | 1.7 | 20 | 2.2 | 1.5 | 0.5 |
| 146 | 146 | 25.6 | 1.1 | 37 | 3.5 | 11.4 | 2.4 |
| 158 | 158 | 43.6 | 1.7 | 51 | 7.5 | 7.4 | 5.8 |
| 162 | 162 | 48.2 | 2.2 | 51 | 6.5 | 2.8 | 4.3 |
| 183 | 183 | 46.7 | 0.6 | 53 | 4 | 6.3 | 3.4 |
| μ |  | | | | | 7.4 | 3.7 |
| σ |  | | | | | 4.2 | 2.8 |
| RMSD |  | | | | | 8.5 | 4.7 |
| R |  | | | | | 0.91 | 0.40 |

Values are the average (μ) and standard deviation (σ) of inter-Cβ distance distributions for double mutants (AA_1_ and AA_2_) of MSBA in the AMP-PNP bound state as calculated from the best 100 Rosetta models according to score and inter-spin label distance distributions from EPR experiment, respectively. The deviation of Rosetta from experiment in terms μ and σ is also given for each double mutant. The bottom four rows show the mean deviation, standard deviation of the deviation, RMSD, and the correlation coefficient (R) of Rosetta with experiment.
