# Supplementary material for: RosettaEPR: Rotamer Library for Spin Label Structure and Dynamics
Source: PLoS One. 2013 Sep 5;8(9):e72851. doi: 10.1371/journal.pone.0072851 (PMC3764097; doi:10.1371/journal.pone.0072851)
Supplement: Table S10 — Analysis of the best ensemble of Rosetta models fitted to the experimental distance probability distributions for T4 lysozyme. (DOC) [file pone.0072851.s025.doc]

**Supplemental Table 1.** Analysis of the best ensemble of Rosetta models fitted to the experimental distance probability distributions for T4 lysozyme.

| AA_1_ | AA_2_ | $\mu_{Rosetta}^{fitted}$ | $\sigma_{Rosetta}^{fitted}$ | μ _EPR_ | σ _EPR_ | $\left\vert\mu_{Rosetta}^{fitted}-\mu_{EPR}\right\vert$ | $\left\vert\sigma_{Rosetta}^{fitted}-\sigma_{EPR}\right\vert$ |
| --- | --- | --- | --- | --- | --- | --- | --- |
| 59 | 159 | 41.8 | 2.7 | 41.9 | 2.7 | 0.1 | 0.0 |
| 60 | 90 | 38.2 | 3.7 | 37.8 | 4.5 | 0.4 | 0.8 |
| 60 | 94 | 25.5 | 3.1 | 25.5 | 3.1 | 0.0 | 0.0 |
| 60 | 109 | 35.2 | 2.5 | 35.2 | 2.6 | 0.0 | 0.1 |
| 60 | 154 | 34.1 | 2.0 | 34.1 | 2.0 | 0.0 | 0.0 |
| 61 | 128 | 46.2 | 2.1 | 46.2 | 2.4 | 0.0 | 0.3 |
| 61 | 135 | 43.8 | 1.6 | 47.2 | 2.2 | 3.4 | 0.6 |
| 62 | 109 | 29.5 | 2.9 | 29.5 | 2.7 | 0.0 | 0.2 |
| 62 | 123 | 42.8 | 2.8 | 42.3 | 3.3 | 0.5 | 0.5 |
| 62 | 134 | 42.1 | 0.0 | 41.1 | 1.5 | 1.0 | 1.5 |
| 62 | 155 | 41.2 | 1.5 | 41.2 | 1.5 | 0.0 | 0.0 |
| 64 | 122 | 34.0 | 2.4 | 34.1 | 2.5 | 0.1 | 0.1 |
| 65 | 76 | 23.2 | 2.7 | 21.4 | 2.8 | 1.8 | 0.1 |
| 65 | 135 | 45.4 | 2.2 | 46.3 | 2.2 | 0.9 | 0.0 |
| 82 | 94 | 29.9 | 2.3 | 30.7 | 3.3 | 0.8 | 1.0 |
| 82 | 132 | 26.5 | 3.3 | 26.3 | 3.5 | 0.2 | 0.2 |
| 82 | 134 | 33.9 | 2.6 | 33.9 | 3.2 | 0.0 | 0.6 |
| 82 | 155 | 35.8 | 2.0 | 35.8 | 2.5 | 0.0 | 0.5 |
| 83 | 123 | 20.5 | 3.3 | 20.5 | 3.4 | 0.0 | 0.1 |
| 83 | 155 | 32.6 | 2.2 | 32.8 | 3.0 | 0.2 | 0.8 |
| 93 | 108 | 23.1 | 3.0 | 23.3 | 4.1 | 0.2 | 1.1 |
| 93 | 112 | 26.1 | 1.3 | 26.1 | 1.5 | 0.0 | 0.2 |
| 93 | 123 | 24.9 | 2.2 | 24.8 | 2.3 | 0.1 | 0.1 |
| 93 | 134 | 29.1 | 2.2 | 29.1 | 2.4 | 0.0 | 0.2 |
| 93 | 154 | 25.0 | 2.1 | 25.1 | 2.4 | 0.1 | 0.3 |
| 94 | 123 | 24.0 | 2.6 | 24.0 | 2.6 | 0.0 | 0.0 |
| 94 | 132 | 31.7 | 1.1 | 31.7 | 1.3 | 0.0 | 0.2 |
| 108 | 123 | 27.6 | 2.4 | 27.6 | 2.4 | 0.0 | 0.0 |
| 108 | 134 | 32.5 | 1.1 | 32.4 | 1.2 | 0.1 | 0.1 |
| 108 | 155 | 35.3 | 1.9 | 35.2 | 2.3 | 0.1 | 0.4 |
| 109 | 134 | 30.4 | 2.0 | 30.6 | 2.8 | 0.2 | 0.8 |
| 115 | 155 | 27.9 | 1.9 | 28.2 | 2.4 | 0.3 | 0.5 |
| 116 | 134 | 20.2 | 1.4 | 20.2 | 1.5 | 0.0 | 0.1 |
| 119 | 128 | 20.0 | 2.3 | 19.9 | 2.3 | 0.1 | 0.0 |
| 119 | 131 | 22.4 | 2.3 | 22.3 | 2.7 | 0.1 | 0.4 |
| 123 | 131 | 22.4 | 2.6 | 22.3 | 2.7 | 0.1 | 0.1 |
| 128 | 155 | 20.4 | 2.6 | 20.7 | 3.7 | 0.3 | 1.1 |
| 140 | 151 | 21.3 | 1.4 | 22.2 | 3.3 | 0.9 | 1.9 |
| μ |  | | | | | 0.3 | 0.4 |
| σ |  | | | | | 0.6 | 0.4 |
| RMSD |  | | | | | 0.7 | 0.6 |
| R |  | | | | | 0.996 | 0.80 |

The average (μ) and standard deviation (σ) of inter-spin label distance distributions for double mutants (AA_1_ and AA_2_) of t4-lysozyme as calculated from the best ensemble of Rosetta models fitted to the experimental distance probability distribution. This fitted μ and σ is compared with μ and σ from experiment. The deviation of Rosetta from experiment in terms μ and σ is also given for each double mutant. The bottom four rows show the mean deviation, standard deviation of the deviation, RMSD, and the correlation coefficient (R) of Rosetta with experiment.
