# Supplementary material for: RosettaEPR: Rotamer Library for Spin Label Structure and Dynamics
Source: PLoS One. 2013 Sep 5;8(9):e72851. doi: 10.1371/journal.pone.0072851 (PMC3764097; doi:10.1371/journal.pone.0072851)
Supplement: Table S11 — Analysis of the best ensemble of Rosetta models fitted to the experimental distance probability distributions for MSBA in the apo open state. (DOC) [file pone.0072851.s026.doc]

**Supplemental Table 1.** Analysis of the best ensemble of Rosetta models fitted to the experimental distance probability distributions for MSBA in the apo open state.

| AA_1_ | AA_2_ | $\mu_{Rosetta}^{fitted}$ | $\sigma_{Rosetta}^{fitted}$ | μ _EPR_ | σ _EPR_ | $\left\vert\mu_{Rosetta}^{fitted}-\mu_{EPR}\right\vert$ | $\left\vert\sigma_{Rosetta}^{fitted}-\sigma_{EPR}\right\vert$ |
| --- | --- | --- | --- | --- | --- | --- | --- |
| 42 | 42 | 42.3 | 9.3 | 36.0 | 10.0 | 6.3 | 0.7 |
| 43 | 43 | 38.3 | 5.9 | 35.0 | 2.5 | 3.3 | 3.4 |
| 142 | 142 | 53.1 | 7.7 | 54.0 | 7.0 | 0.9 | 0.7 |
| 143 | 143 | 50.4 | 9.9 | 54.0 | 5.6 | 3.6 | 4.3 |
| 144 | 144 | 35.1 | 4.0 | 35.0 | 2.5 | 0.1 | 1.5 |
| 146 | 146 | 42.5 | 1.0 | 42.0 | 3.8 | 0.5 | 2.8 |
| 158 | 158 | 39.3 | 2.2 | 36.0 | 1.5 | 3.3 | 0.7 |
| 162 | 162 | 43.7 | 5.6 | 44.0 | 3.2 | 0.3 | 2.4 |
| 183 | 183 | 44.0 | 7.0 | 43.0 | 13.2 | 1.0 | 6.2 |
| μ |  | | | | | 2.1 | 2.5 |
| σ |  | | | | | 2.0 | 1.8 |
| RMSD |  | | | | | 2.9 | 3.1 |
| R |  | | | | | 0.95 | 0.59 |

The average (μ) and standard deviation (σ) of inter-spin label distance distributions for double mutants (AA_1_ and AA_2_) of MSBA in the apo open state as calculated from the best ensemble of Rosetta models fitted to the experimental distance probability distribution. This fitted μ and σ is compared with μ and σ from experiment. The deviation of Rosetta from experiment in terms μ and σ is also given for each double mutant. The bottom four rows show the mean deviation, standard deviation of the deviation, RMSD, and the correlation coefficient (R) of Rosetta with experiment.
