# Supplementary material for: RosettaEPR: Rotamer Library for Spin Label Structure and Dynamics
Source: PLoS One. 2013 Sep 5;8(9):e72851. doi: 10.1371/journal.pone.0072851 (PMC3764097; doi:10.1371/journal.pone.0072851)
Supplement: Table S14 — Disagreement to experimental distance distributions of models selected by score and fitting for MsbA in the apo-open state double mutant models. (DOC) [file pone.0072851.s029.doc]

**Supplemental Table 1.** Disagreement to experimental distance distributions of models selected by score and fitting for MsbA in the apo-open state double mutant models.

| AA_1_ | AA_2_ | Top 200 Disagreement | Fitted  Disagreement | Size | Percent Disagreement Reduction |
| --- | --- | --- | --- | --- | --- |
| 42 | 42 | 0.209 | 0.103 | 8 | 50.9 |
| 43 | 43 | 0.188 | 0.039 | 9 | 79.2 |
| 142 | 142 | 0.107 | 0.065 | 12 | 39.6 |
| 143 | 143 | 0.135 | 0.055 | 16 | 58.9 |
| 144 | 144 | 0.333 | 0.040 | 18 | 88.0 |
| 146 | 146 | 0.348 | 0.176 | 3 | 49.4 |
| 158 | 158 | 0.266 | 0.080 | 4 | 70.0 |
| 162 | 162 | 0.072 | 0.022 | 17 | 68.8 |
| 183 | 183 | 0.139 | 0.070 | 24 | 49.7 |
| μ |  | 0.200 | 0.072 | 12 | 61.6 |
| σ |  | 0.093 | 0.043 | 7 | 15.0 |

For MsbA in the apo-open state, the cumulative Euclidean disagreement values of the best 200 relaxed structures by Rosetta score (Top 200 Disagreement) and an ensemble of Rosetta models selected to fit the experimental (Fitted Disagreement). The disagreement is calculated between the distance distribution obtained from the Rosetta models and the corresponding experimental distance distribution, with 0 being perfect agreement. The size of the fitted ensemble is also provided (Size). The amount that the disagreement is reduced as a percentage of starting disagreement (Percent Disagreement Reduction) is calculated as $\left(Top200Disgreement-FittedDisagreement\right)/Top200Disagreement* 100$
