# Supplementary material for: RosettaEPR: Rotamer Library for Spin Label Structure and Dynamics
Source: PLoS One. 2013 Sep 5;8(9):e72851. doi: 10.1371/journal.pone.0072851 (PMC3764097; doi:10.1371/journal.pone.0072851)
Supplement: Table S16 — The MMM and MTSSLWizard average (μ) and standard deviation (σ) of inter-spin label distance distributions for double mutants of T4 lysozyme. (DOC) [file pone.0072851.s031.doc]

| **Supplemental Table 16.** The MMM and MTSSLWizard average (μ) and standard deviation (σ) of inter-spin label distance distributions for double mutants of T4 lysozyme. | | | | | |
| --- | --- | --- | --- | --- | --- |
| AA_1_ | AA_2_ | μ _MTSSLWizard_ | σ _MTSSLWizard_ | μ _MMM_ | σ _MMM_ |
| 59 | 159 | 42.1 | 2.8 | 38.2 | 3.5 |
| 60 | 90 | 45.4 | 2.5 | 43.3 | 3.1 |
| 60 | 94 | 32.4 | 3.4 | 33.4 | 3.8 |
| 60 | 109 | 39.0 | 3.2 | 34.3 | 4.2 |
| 60 | 154 | 38.5 | 2.9 | 39.7 | 3.4 |
| 61 | 80 | 33.9 | 3.7 | 31.1 | 4.8 |
| 61 | 86 | 44.6 | 3.3 | 40.5 | 3.7 |
| 61 | 128 | 53.0 | 2.6 | 49.1 | 2.6 |
| 61 | 135 | 50.4 | 2.3 | 45.7 | 2.8 |
| 62 | 109 | 34.9 | 3.5 | 30.6 | 4.2 |
| 62 | 123 | 51.2 | 2.0 | 48.2 | 2.4 |
| 62 | 134 | 48.9 | 2.8 | 45.5 | 2.0 |
| 62 | 155 | 46.2 | 1.7 | 42.6 | 2.2 |
| 64 | 122 | 38.4 | 2.2 | 37.6 | 3.7 |
| 65 | 76 | 18.2 | 3.1 | 17.7 | 4.0 |
| 65 | 80 | 24.7 | 3.4 | 22.8 | 4.7 |
| 65 | 86 | 36.9 | 2.9 | 33.4 | 3.7 |
| 65 | 135 | 45.9 | 2.5 | 41.9 | 3.0 |
| 80 | 135 | 34.4 | 3.3 | 32.4 | 4.0 |
| 82 | 94 | 31.9 | 2.6 | 28.5 | 3.1 |
| 82 | 132 | 27.0 | 3.0 | 24.7 | 4.5 |
| 82 | 134 | 36.2 | 2.4 | 30.8 | 3.4 |
| 82 | 155 | 37.5 | 3.0 | 33.0 | 2.6 |
| 83 | 123 | 17.7 | 4.5 | 17.7 | 4.0 |
| 83 | 155 | 33.8 | 2.6 | 29.6 | 3.9 |
| 86 | 112 | 19.6 | 2.5 | 16.3 | 3.5 |
| 86 | 119 | 13.7 | 4.0 | 16.0 | 3.5 |
| 88 | 100 | 5.6 | 1.7 | 11.6 | 1.1 |
| 89 | 93 | 16.8 | 3.7 | 17.5 | 3.8 |
| 89 | 96 | 11.4 | 3.0 | 16.4 | 3.3 |
| 93 | 108 | 28.1 | 3.2 | 26.4 | 4.5 |
| 93 | 112 | 32.2 | 2.5 | 29.4 | 2.7 |
| 93 | 123 | 26.0 | 3.0 | 24.1 | 2.9 |
| 93 | 134 | 34.4 | 2.3 | 28.5 | 3.2 |
| 93 | 154 | 26.3 | 2.3 | 21.2 | 3.3 |
| 94 | 123 | 24.8 | 2.6 | 21.9 | 3.7 |
| 94 | 132 | 32.3 | 1.5 | 26.8 | 3.4 |
| 108 | 123 | 31.5 | 2.2 | 26.1 | 3.3 |
| 108 | 134 | 33.2 | 1.5 | 29.4 | 1.8 |
| 108 | 155 | 36.9 | 1.6 | 31.9 | 2.8 |
| 109 | 134 | 30.6 | 2.7 | 26.7 | 2.9 |
| 115 | 155 | 33.0 | 2.2 | 26.4 | 2.9 |
| 116 | 134 | 18.0 | 2.5 | 17.1 | 3.4 |
| 119 | 128 | 16.8 | 3.5 | 14.9 | 3.6 |
| 119 | 131 | 23.1 | 3.3 | 18.1 | 3.9 |
| 120 | 131 | 12.4 | 2.9 | 13.6 | 2.0 |
| 123 | 131 | 24.2 | 2.7 | 18.8 | 3.7 |
| 127 | 151 | 14.6 | 2.9 | 16.0 | 3.5 |
| 127 | 154 | 9.0 | 2.6 | 13.0 | 2.4 |
| 127 | 155 | 10.2 | 3.4 | 14.0 | 2.6 |
| 128 | 155 | 19.0 | 3.1 | 16.0 | 3.3 |
| 131 | 150 | 8.3 | 3.0 | 13.8 | 2.7 |
| 134 | 151 | 15.1 | 2.3 | 14.3 | 2.9 |
| 140 | 151 | 21.8 | 3.2 | 18.7 | 4.2 |
