# Supplementary material for: RosettaEPR: Rotamer Library for Spin Label Structure and Dynamics
Source: PLoS One. 2013 Sep 5;8(9):e72851. doi: 10.1371/journal.pone.0072851 (PMC3764097; doi:10.1371/journal.pone.0072851)
Supplement: Table S17 — Descriptions of the disagreement between prediction and experiment for the average distance and standard deviation of distance distributions from RosettaEPR, MMM, and MTSSLWizard. (DOC) [file pone.0072851.s032.doc]

| Supplemental Table 1. Descriptions of the disagreement between prediction and experiment for the average distance and standard deviation of distance distributions from RosettaEPR, MMM, and MTSSLWizard. | | | | | | |
| --- | --- | --- | --- | --- | --- | --- |
|  | Average Distance | | | Standard Deviation | | |
|  | Rosetta | MMM | MTSSLWizard | Rosetta | MMM | MTSSLWizard |
| Mean Absolute Error | 3.5 | 3.5 | 3.0 | 0.9 | 1.0 | 0.7 |
| RMSD | 4.5 | 4.0 | 3.9 | 1.1 | 1.2 | 0.9 |
| Standard Error* | 4.6 | 3.9 | 3.2 | 0.9 | 0.7 | 0.6 |
| *calculated using Excel STEYX function | | | | | | |

Slide 1: The average distance of predicted distance distributions compared to experimental distance distributions. Shown are results for 54 double mutants of T4-lysozyme.

Slide 2: The standard deviation of predicted distance distributions compared to experimental distance distributions. Shown are results for 54 double mutants of T4-lysozyme. Note that MTSSLWizard and MMM are comparable with RosettaEPR in terms of error and RMSD. However, RosettaEPR correlates much better with experiment, with the prediction range being compressed for MTSSLWizard and MMM as compared to RosettaEPR.
