# Supplementary material for: RosettaEPR: Rotamer Library for Spin Label Structure and Dynamics
Source: PLoS One. 2013 Sep 5;8(9):e72851. doi: 10.1371/journal.pone.0072851 (PMC3764097; doi:10.1371/journal.pone.0072851)
Supplement: Experimental Procedures S1 — Command lines used for Rosetta protocols. (DOC) [file pone.0072851.s033.doc]

Supplemental Experimental Procedures

The command line arguments used for the Rosetta relaxation protocol (“relax”) include:

relax.linuxgccrelease -database minirosetta_database/ -in:file:s start_structure.pdb -out:file:fullatom -in:file:extra_res_fa R1A.params -out:prefix m_142_ -run::constant_seed -run::jran $seed_number -nstruct 10

The command line arguments used for the Rosetta relaxation protocol with membrane potentials include:

relax.linuxgccrelease -database rosetta_database/ -in:file:s start_structure.pdb -out:file:fullatom -in:file:extra_res_fa R1A.params -out:prefix m_18_ -run::constant_seed -run::jran $seed_number -nstruct 10 -relax:membrane -score:weights membrane_highres.wts -membrane:normal_cycles 100 -membrane:normal_mag 15 -membrane:center_mag 2 -file:spanfile msba.span

The command line arguments used for the Rosetta fixed backbone design protocol (“fixbb”) include :

fixbb.linuxgccdebug -database minirosetta_database/ -in:file:s pseudo_wildtype.pdb -out:file:fullatom -in:file:extra_res_fa R1A.params -resfile resfile.pack -out:prefix create_start_structure/

An example of the contents of one of the residue mutation files (“resfile”) used to create a T4 lysozyme double mutant is :

NATRO

start

059 A EMPTY R1A

159 A EMPTY R1A

Supplemental Figure 1 Heat maps for 58 double mutants of T4 lysozyme showing Gaussian distributions given by experimentally measured mean and standard deviation parameters compared with distance distributions recovered by Rosetta from the top 200 models according to Rosetta score. *Experimental* distance distributions are the *top bar* and Rosetta distributions are the bottom bar for each pair of heat maps. Distances are given in Angstroms, and the probability of observing a distance is defined by grayscale. Mutants 131/154, 131/151, 140/147, 116/131 were excluded from statistical analysis but are shown here for completeness.

**Supplemental Figure 1** continued.
